# Additional Evidence for Morpho-Dimensional Tooth Crown Variation in a New Indonesian *H. erectus* Sample from the Sangiran Dome (Central Java)

**DOI:** 10.1371/journal.pone.0067233

**Published:** 2013-07-03

**Authors:** Clément Zanolli

**Affiliations:** 1 Multidisciplinary Laboratory, International Centre for Theoretical Physics, Trieste, Italy; 2 Département de Préhistoire, UMR 7194, MNHN Paris, France; Museo Nazionale Preistorico Etnografico ‘L. Pigorini’, Italy

## Abstract

This contribution reports fifteen human fossil dental remains found during the last two decades in the Sangiran Dome area, in Central Java, Indonesia. Among this sample, only one of the specimens had already been briefly described, with the other fourteen remaining unreported. Seven of the fifteen isolated teeth were found in a secured stratigraphic context in the late Lower-early Middle Pleistocene Kabuh Formation. The remaining elements were surface finds which, based on coincidental sources of information, were inferred as coming from the Kabuh Formation. Mainly constituted of permanent molars, but also including one upper incisor and one upper premolar, this dental sample brings additional evidence for a marked degree of size variation and time-related structural reduction in Javanese *H. erectus*. This is notably expressed by a significant decrease of the mesiodistal diameter, frequently associated to the reduction or even loss of the lower molar distal cusp (hypoconulid) and to a more square occlusal outline. In addition to the hypoconulid reduction or loss, this new sample also exhibits a low frequency of the occlusal Y-groove pattern, with a dominance of the X and, to a lesser extent, of the+patterns. This combination is rare in the Lower and early Middle Pleistocene paleoanthropological record, including in the early Javanese dental assemblage from the Sangiran Dome. On the other hand, similar dental features are found in Chinese *H. erectus* and in *H. heidelbergensis*. As a whole, this new record confirms the complex nature of the intermittent exchanges that occurred between continental and insular Southeast Asia through the Pleistocene.

## Introduction

Since the first discovery of *Pithecanthropus erectus* by E. Dubois [1], more than 230 fossil hominid dental elements [2]−[20] have been found in Java. Most of the specimens originating from the Lower-Middle Pleistocene Kabuh (Bapang) Formation are undisputedly attributed to *H. erectus* because of their evident derived human morphology (rev. in [13]). However, controversial historical discussions arose regarding the taxonomic and phylogenetic affinities of the archaic Lower Pleistocene Javanese hominid dentognathic remains from the Pucangan (Sangiran) Formation [4], [8], . This situation is mainly due to the morpho-dimensional variability of the Javanese specimens together with the homoplastic dental features noticed between Lower Pleistocene *Homo* and *Pongo* [4], [8−13], [16], . Consequently, some fossils like, for example, Sangiran 4, Sangiran 5 or Sangiran 6a, have been tentatively attributed to other taxa (*e*.*g*., *Meganthropus paleojavanicus*, *Pithecanthropus dubius* or even *Pongo*), or are still pending attribution [8], . This taxonomic incertitude probably relates to the impact in terms of evolutionary dynamics of the eustatic variations which have cyclically affected the Indonesian archipelago during the Quaternary. Sea level variations drastically modified the paleogeography of the region, allowing the formation of temporary land-bridges between continental and insular domains and, therefore, intermittent exchanges with the Asian mainland. In this dynamic scenario, it is likely that isolation phases have periodically shaped the local biodiversity, including *H. erectus* [45]−[47].

Since the late 1980s, a Franco-Indonesian collaboration between the Muséum national d’Histoire Naturelle of Paris and the Indonesian Archeological Services has conducted survey and excavations of numerous sites in the Sangiran Dome area (Central Java, Indonesia), allowing a better understanding of the geological [47]−[50] and paleoenvironmental context [51]−[55]. The Sangiran Dome corresponds to a partially eroded anticlinal, characterized by the outcropping of two Early-Middle Pleistocene stratigraphical units bearing human fossils: the Pucangan (Sangiran) and the Kabuh (Bapang) Formations. The Pucangan Formation and the lowest part of the Kabuh Formation, referred as the “Grenzbank Zone”, are usually reported as belonging to the Lower Pleistocene, with a minimal age ranging from >1.5 Ma [Bibr pone.0067233-Larick1] [56−57] to ca. 0.9 Ma [58]−[60]. Conversely, based on the currently available magneto-stratigraphic and radiometric records, the precise chronology of the Kabuh Formation remains a matter of discussion (rev. in [46], [61], being alternatively referred to as the Lower Pleistocene (ca. 1.5−1.0 Ma [56−57], ) or to the late Lower-early Middle Pleistocene (ca. 0.8 to 0.5 Ma; [49−50], [58−59], . After several field seasons over the last two decades on the Sangiran Dome, among hundreds of fossil faunal remains and a few lithic artifacts [48], the Franco-Indonesian team recovered fifteen isolated human teeth coming from excavations, surveys or sporadic finds. This study aims to analyze and comparatively assess this unique fossil dental assemblage.

### Material and Methods

**Figure 1:**
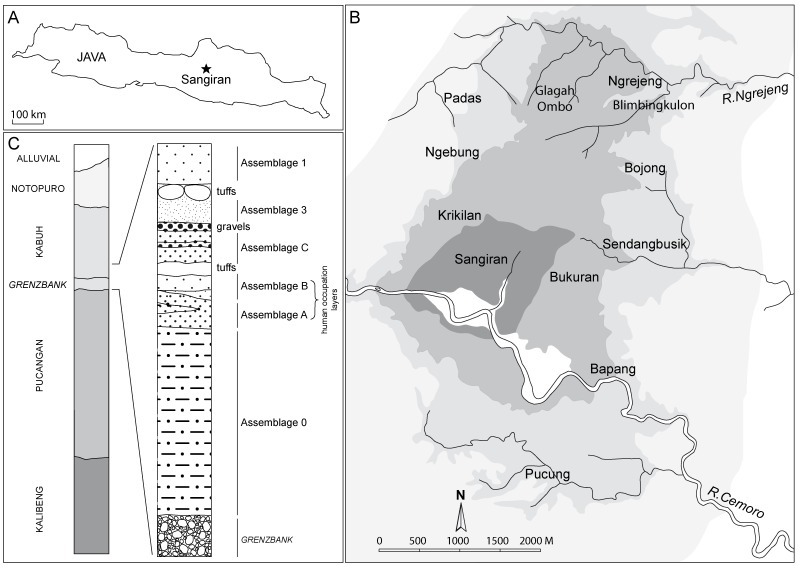
Location of the Sangiran Dome in the island of Java (A) with the distribution of the main geo-paleontological sites having provided human fossil remains (B) and the simplified stratigraphic section of the excavated area at Ngebung. (modified from [14], [50]). The site of Pancuran, near Miri, where the specimens MI92.1 and MI92.2 were recovered is located ca. 10 km North of the Sangiran Dome.

The original human fossil dental sample described here is constituted by fifteen permanent isolated teeth from the Kabuh (Bapang) Formation, all of which are currently stored at the Balai Pelestarian Situs Manusia Purba Sangiran. Two specimens (MI92.1 and MI92.2) were discovered at Pancuran, near Miri, at the North of the Sangiran Dome, in sand deposits correlated to the Kabuh Formation [7], [64]. Among the eleven specimens coming from the Ngebung hills, Northwest of the Sangiran Dome (Figure 1), three were found in stratigraphic context (NG91-G10 n°1, NG92 D6 ZE 57 s/d 76, NG9505) during survey or the excavation of human occupation floors [48], [50], [65] in levels directly connected to the lower part of the Kabuh Formation [50] (Figure 1C). Eight are isolated teeth coming from Ngebung area and represent surface finds. Because of their state of fossilization, the local geological setting and their co-resemblance, including with specimens found in stratigraphic context, the possibility that they come from subrecent levels was clearly rejected. Their original stratigraphic positions were thus reasonably inferred from coincidental sources of information, pointing towards a Kabuh Formation origin. Two teeth were found in stratigraphic context during excavation of the Kabuh Formation, one coming from Padas (PDS0712), Northwestern part of the Sangiran Dome (Figure 1) and one from Pucung (PCG09_KII_Z:1.37), at the South of the Sangiran Dome (Figure 1).

Except for three teeth (NG91-G10 n 1, NG92 D6 ZE 57 s/d 76, PCG09_KII_Z:1.37), no formal label was given yet to the specimens. In order to assign them a designation label, we used the ongoing system combining the name of the village, the date of discovery [12], and adding a dot with a number when many specimens where found at the same place and date.

In the following descriptions, we used abbreviations of anatomical dental terms as follow: BL, buccolingual/ly; C7, tuberculum intermedium; Dmr, distal marginal ridge; DTC, distal trigonid crest; End, entoconid; Fc, central fovea; Fd, distal fovea; Fm, mesial fovea; Hld, hypoconulid; Hy, hypocone; Hyd, hypoconid; ICF, interproximal contact facet; LL, labiolingual/ly; MD, mesiodistal/ly; Me, metacone; Med, metaconid; Mmr, mesial marginal ridge; MTC, middle trigonid crest; Pa, paracone; Pr, protocone; Prd, protoconid. All of the dental elements described below were examined at a macroscopic level and at low magnification (x6.5 and x10). The degree of occlusal wear was assessed following Smith [66] and the references to nonmetric traits followed the Arizona State University Dental Anthropology System (ASUDAS [67]−[68]). Crown size was measured to the nearest 0.1 mm with a Mitutoyo Digimatic caliper. The maximum MD [69] and BL diameters were estimated. The computed crown area (CCA = MD*BL, in mm^2^) and the crown shape index (CI = BL/MD*100) were calculated for the lower molars included in the sample and compared to the variation shown by a selected number of extant and Pleistocene dental samples/specimens (see detail in Table S1), separated in fourteen groups: extant humans (EH), fossil modern humans (FMH), *H. floresiensis* (HF), Neanderthals (NEA), *H. heidelbergensis* (HH) including European (HHE) and North African (HHNA) specimens (*sensu* [70]), *H. antecessor* (HA), Chinese *H. erectus* (HEC), Javanese *H. erectus* (HEJ), robust hominid from Java (RHJ, *sensu* [12]), *H. erectus* from Georgia (HEG), *H. erectus* from East Africa (HEA, *sensu* [71]−[72]), *H. habilis*/*rudolfensis* (HHR, *sensu* [71]−[72]). For the Upper Pleistocene groups, we extracted the dental diameters from the Anthropological Database (anthropologicaldata.free.fr [73]).

Cusp base areas, shown to have significant taxonomic value in discriminating among Pleistocene hominin groups [71], as well as for specific and sub-specific level assessment in extant hominids [81]−[83], were estimated for eight lower molars. Conversely, advanced wear obliterated the occlusal cusp outline of the first molars MI92.1 and NG92.2. Measurements of individual cusps on occlusal photographs followed the procedure established by Bailey [79], by orienting the plane of the cervical line of the molar perpendicularly to the camera objective. Interproximal wear was conservatively corrected, and accessory cusps (e.g., C6 or C7) were subdivided, with equal parts being added to the areas of the adjacent principal cusps [74]−[78]. For comparative purpose, we estimated the cusp base areas for the second and third molar positions of four lower molars of *H. erectus* from Java (Sangiran 1b LM2 & LM3, Sangiran 7−64 LM2, Sangiran 7−65 LM2) and of six *H. heidelbergensis* teeth from Tighenif (Tighenif 1, Tighenif 2, Tighenif 3). In addition to the data available for the second molar [84], we also assessed the cusp base areas of five modern human third molars.

An adjusted Z-score analysis [85]−[86] was performed on the cusp base areas proportions of the lower molars from the new material and on five fossil and extant comparative groups. This statistical method allows the comparison of unbalanced samples, which is often limitative for the fossil record, using the Student’s t inverse distribution following the formula: ((x−m)/(s*sqrt(1+1/n))/(Student.t.inverse(0.05;n−1)), where *x* is the value of the variable (*e*.*g*., Prd area of NG9107.2), *m* is the mean of the same variable for a comparative sample (*e*.*g*., Prd area for EH), *n* is the size of the comparative sample for this variable and *s* is the standard deviation of the comparative sample for this variable. We performed the cusp base areas Z-score analyses separately for the second and third molars, as well as with the mixed sample of molars for the specimens for which the serial position remained uncertain.

### Ethics Statement

This study concerns the analysis of an original fossil human dental sample constituted by fifteen isolated specimens: MI92.1, MI92.2, NG0802.1, NG0802.2, NG0802.3, NG9107.2, NG91-G10 n°1, NG92.1, NG92.2, NG92.3, NG92.4, NG92 D6 ZE 57 s/d 76, NG9505, PCG09_KII_Z:1.37, PDS0712. The specimens are permanently stored at the Balai Pelestarian Situs Manusia Purba Sangiran, Java, Indonesia. F. Sémah, director of the French team, and H. Widianto, curator, have made possible this study within the framework of a long-term scientific collaboration in the fields of geo-prehistory and paleoanthropology between the French MNHN, the Pusat Penelitian Arkeologi of Jakarta, and the Balai Pelestarian Situs Manusia Purba of Sangiran, and D. Grimaud-Hervé has kindly assured the temporary transport in 2009−2010 of some specimens in France for analysis. All necessary permits were obtained for the described study, which complied with all relevant regulations.

## Results

### Descriptions

The individual dimensional characteristics and non-metric features of the investigated specimens are shown in Table 1.

**Table 1:** Individual non-metric crown features and dimensions of the new Javanese fossil human dental sample.

Upper jaw		MI92.2				NG9505					NG91-G10 n°1		PDS0712		NG0802.1			
		URI2				URP3/4					ULM1/2		URM2/3		ULM3			
wear degree		4		wear degree		2−3		wear degree			3		1−2		1			
labial curve		1		tri-cusped premolars		0		metacone			4		3−4		–			
shoveling		2−3?		distosagittal ridge		0		hypocone			5		4		3			
double-shoveling		2		enamel hypoplasia		0		cusp 5			–		0		0			
interruption groove		D		carious lesion		0		Carabelli’s trait			–		0		2			
tuberculum dentale		–		MD (mm)		7.5		parastyle			0		0		0			
enamel hypoplasia		1		BL (mm)		9.5		enamel extension			1		1		0			
carious lesion		0						enamel hypoplasia			0		1		0			
MD (mm)		8.1						carious lesion			0		0		0			
BL (mm)		7.0						MD (mm)			12.1		9.9		9.0			
								BL (mm)			12.2		12.2		11.2			
**Lower jaw**	**MI92.1**		**NG92.2**		**NG92.1**		**NG92.4**		**NG0802.3**	**PCG09_KII_Z:1.37**		**NG92.3**		**NG92 D6 ZE 57 s/d 76**		**NG0802.2**		**NG9107.2**
	**LRM1**		**LLM1**		**LRM2**		**LRM2**		**LRM2**	**LLM2**		**LLM2/3**		**LRM2/3**		**LRM2/3**		**LLM3**
wear degree	4		4		4		4		1	1		2		1		1		3
groove pattern	Y		+		X		+		X	X		+		X		X		Y
anterior fovea	–		–		–		–		1	1		2		2		1		–
cusp number	5		5		4		4		4	4		5		4		6		5
mid-trigonid crest	–		–		1?		–		1A	1A		1A		1A		1A		1A
distal trigonid crest	–		–		–		–		0	0		0?		0		0		0
deflecting wrinkle	–		–		–		–		0	0		2?		0		0		3
protostylid	–		–		–		–		0	0		0		1		0		-
cusp 5	3?		4		0		0		0	0		3−4		0		4−5		4
cusp 6	–		–		–		–		0	0		–		0		2		0
cusp 7	–		–		–		3		0	0		0		0		0		0
enamel extension	0		0		1		0		1	0		1		1		2		1
enamel hypoplasia	0		0		1		1		1	1		0		1		0		1
carious lesion	0		0		0		0		0	0		0		0		0		0
MD (mm)	12.0		11.2		10.5		10.1		10.8	10.1		11.8		11.0		11.3		10.9
BL (mm)	11.6		10.8		10.2		9.5		10.1	10.0		11.0		10.8		10.7		10.1

### Upper Incisor

**Figure 2:**
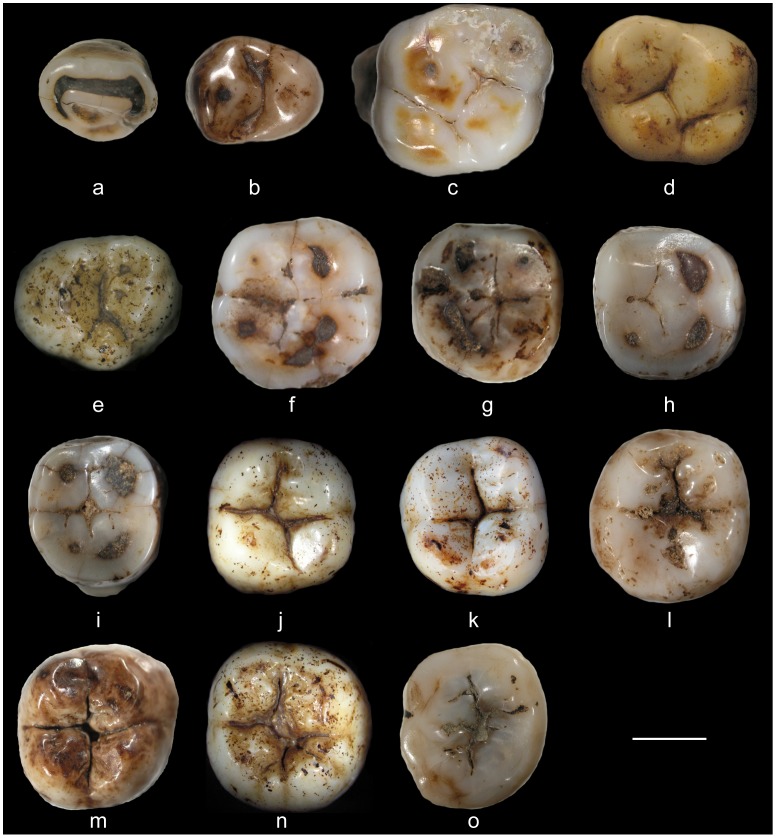
The fifteen permanent crowns forming the new Javanese fossil human dental sample from the Sangiran Dome in occlusal view. : a, MI92.2; b, NG9505; c, NG91-G10 n°1; d, PDS0712; e, NG0802.1; f, MI92.1; g, NG92.2; h, NG92.1; i, NG92.4; j, NG0802.3; k, PCG09_KII_Z:1.37; l, NG92.3, m, NG92 D6 ZE 57 s/d 76; n, NG0802.2; o, NG9107.2. Scale is 5 mm.

MI92.2 is an upper right lateral incisor (URI2) crown with a root fragment (Figure 2a & Figure S1). The enamel, beige to light brown, presents a slight depression 3 mm above the cervical margin at the centre of the mesial half of the labial aspect. A shallow but large enamel hypoplasia is found around half of the labial aspect. The crown is relatively worn (stage 4), showing a large dentine exposure with a still complete enamel ring and an incisal edge LL oriented. The shoveling and double shoveling are quite marked. An interruption groove cuts the distal marginal crest. The mesial and distal ICF are ovoid and concave and situated in the upper part of their respective aspects. The dark etched root fragment, broken transversally 7 mm under the crown, does not exhibit longitudinal grooves. In the inferior view, the root has an ovoid outline and the pulp canal is clearly visible.

### Upper Premolar

NG9505 is an upper right third or fourth premolar crown (URP3/4) with a root fragment (Figure 2b & Figure S2). The enamel, ivory to brown, does not show any alteration. Moderately worn (stage 2−3), the crown exhibits a small dentine exposure on the Pa. Two cusps of similar area are represented, with the Pa being higher than the Pr. The MD groove is clearly marked and uninterrupted from the Fm to the Fd. The bulging and thick Mmr is slightly incised while the thinner and lower Dmr is complete. In lateral view, the distal, buccal and lingual aspects of the crown are vertically set while the mesial one tilts occlusally. A small, circular and flat ICF is located in the middle of the mesial face. On the other hand, the distal ICF, which is larger, more elongated and concave, follows the occlusal margin. The dark root fragment, broken 5 mm under the crown, presents a longitudinal groove on the mesial and distal sides. In the inferior view, a buccal and a lingual pulp canal are visible.

### Upper Molars

NG91-G10 n°1 represents an isolated upper left first or second molar (ULM1/2) with the complete crown and fragmentary roots (Figure 2c & Figure S3). The well preserved ivory enamel shows no alterations related to post-depositional taphonomic factors or enamel hypoplasic defects. The crown, rhomboidal in the occlusal view, is high, even if moderately worn (stage 3), with smoothed lingual cusps, slightly more elevated buccal cusps, and two small dentine spots on the Pa and Pr. Four main cusps are visible with Pr >Pa>Hy = Me. On the lingual aspect of the Pr, a slight protuberance indicates the presence of a Carabelli cusp, but, due to the occlusal wear, its size and morphology cannot be scored. The Mmr, despite being affected by occlusal wear, is thick and complete, while the Dmr, still high, is thick and slightly incised. The transverse crest is not very elevated, thick or complete, while the oblique crest is thicker and incised by the central groove. The Fm is erased by the wear, but the triangular Fc and the narrow Fd are still perceptible. In the lateral view, the mesial and distal crown faces diverge occlusally, the lingual aspect is bulging and the buccal face slopes towards the cervix. A large, ovoid and flat ICF is located on the occlusobuccal quarter of the mesial aspect. The distal ICF, also large, ovoid and flat, is situated in the middle of the distal aspect. The light brown root fragment shows two buccal roots and one lingual, the two former broken 3 mm under the crown and identified by two distinct pulp canals. The lingual canal preserves 10 mm of its length and exhibits a small pulp canal. The buccal roots were broken before a possible bifurcation, precluding any identification of their morphology. A longitudinal furrow runs along the lingual root from under the cervical margin of the crown down to the fracture.

This specimen was previously described as belonging to *H. erectus* [7], [87].

PDS0712 corresponds to an upper right second or third molar crown (URM2/3) with a short root fragment (Figure 2d & Figure S4). The enamel, ivory with orange spots, shows a slight cervical hypoplasia. The crown, displaying a trapezoidal outline in occlusal view, reveals little attrition (stage 1−2) with large occlusal facets converging to the Fc but still high and slightly smoothed cusps. Four main cusps are developed following the sequence Pr >Pa>Me>Hy. The Mmr is thick, low and complete while the Dmr is much thinner, lower and incomplete. There is no apparent transverse crest while a thick and very low oblique crest is cut by the deep central groove. In the lateral view, the mesial and distal aspects are relatively flat and diverging occlusally, while the buccal and lingual faces are convex and diverging cervically. A small, circular and flat ICF is located in the occlusobuccal quarter of the mesial aspect while no distal ICF is present. The dark root fragment is broken irregularly 1 mm under the cervix. In the inferior view, the pulp cavity is visible, with four horns corresponding to each cusp.

NG0802.1 is an upper left third molar crown (ULM3) with a broken root fragment (Figure 2e & Figure S5). The ivory enamel is slightly etched, but displays no structural anomaly. The crown, ovoid in the occlusal view, is virtually unworn (stage 1). Five cusps are visible, with the Pr being the largest one, the Pa and Me being fused together with only two small apices visible. A reduced Hy is placed distally and a small protoconule is present. A Carabelli’s pit is located on the external aspect of the Pr. The Mmr is thick, low and complete, while the Dmr is thick and incised. The transverse and oblique crests are only slightly perceptible on the buccal cusps. The Fm is small, ovoid and shallow, while the Fd is not well delimited. In lateral view, the mesial, distal and buccal aspects of the crown are convex and vertically set, while the lingual face is convex and sloping cervically. A small, circular and flat ICF is situated in the lingual half of the mesial face and no distal ICF is present. The dark etched root fragment, broken 5 mm under the cervix on the buccal side and 9 mm under on the lingual side, shows three branches: a mesio-buccal, a mesio-lingual and a distal. In the inferior view, three pulp canals are visible, with the distal one being filled with sediment.

### Lower Molars

MI92.1 is a lower right first molar crown (LRM1) with a short root fragment (Figure 2f & Figure S6). The enamel, ivory to light brown, shows no structural anomaly. The crown, displaying a pentagonal outline in occlusal view, is relatively worn (stage 4), with dentine patches on the flattened buccal cusps and on the End while the higher Med only shows a small spot of dentine exposure. Five cusps are present according to the sequence Prd>Med ≥ End>Hyd>Hld. Despite the advanced occlusal wear degree, a Y groove pattern is still discernible, while all crests and fovea were erased by the attrition. In buccal view, the lateral aspects are slightly convex and vertical. The mesial and distal ICF are ovoid, slightly concave and elongated, situated in the occlusal half of their respective aspects. The dark root fragment has two branches, a buccal and a lingual, the first broken just under the cervix while the second is broken 3 mm below. In the inferior view, the pulp cavity is visible, showing four horns corresponding to the main cusps, except that of the Hld.

NG92.2 is a lower left first molar crown (LLM1) with a root fragment (Figure 2g & Figure S7). The enamel, beige and brown, does not show any structural anomaly. The enamel is well preserved even if a small chip was broken between the Prd and the Hyd. The crown, presenting a nearly square outline in occlusal view, is relatively worn (stage 4), only the Med remaining slightly elevated even if very smoothed, while the other cusps are completely flat. Five main cusps are visible following the pattern Prd>Med>End>Hyd>Hld. The occlusal grooves are very attenuated by wear, but it is still possible to detect a+pattern. Conversely, the crests and fovea were removed by the wear. In lateral view, the mesial, distal and buccal crown faces are slightly convex while the lingual aspect is bulging. A wide, ovoid and concave ICF occupies nearly all of the mesial face. On the buccal half of the distal aspect, an extended, circular and concave ICF is present. The brown root fragment shows two branches, mesial and distal, both BL elongated, the distal one broken 7 mm under the cervix. No longitudinal furrow can be discerned. In inferior view, the pulp canals are filled with sediment.

NG92.1 is a lower right second molar crown (LRM2) with a small root fragment (Figure 2h & Figure S8). The ivory enamel exhibits a slight hypoplasia all around the cervix. The crown, presenting a square outline, is relatively worn (stage 4), with only the Med remaining elevated, even if very smoothed, the other cusps are completely flat and present moderately developed dentine patches. Four main cusps are visible: Prd>Hyd>End>Med. Even considering the advanced degree of occlusal wear, an X groove pattern is still perceptible, while most of the occlusal relief was erased. Nonetheless, remnants of a non-scorable MTC are still discernible. In the lateral view, all crown aspects are relatively flat and vertical. Large, elongated and slightly concave mesial and distal ICFs are present in the center of the superior half of their respective face. The root fragment is broken 4 mm under the cervix on the lingual side and 2 mm under on the buccal side. In the inferior view, the pulp cavity is visible, showing four horns corresponding to each cusp.

NG92.4 is a lower right second molar crown (LRM2) with a short root fragment (Figure 2i & Figure S9). The enamel, ivory to beige, presents a slight hypoplasia encircling the cervix. The crown, showing a sub-squared outline in occlusal view, is relatively worn (stage 4), with flattened relief and dentine spots on each main cusp. Four main cusps are identifiable, as well as a well-developed C7, following the pattern Prd>Hyd>Med ≥ End>C7. Despite the advanced wear degree, a+groove pattern is still obvious, while the other occlusal reliefs were erased by the attrition. In lateral view, the mesial, distal, and lingual crown aspects are flat and vertical while the buccal face is flat and slopes cervically. Large, ovoid and flat ICF are located in the middle of the superior half of the mesial and distal aspects. The dark mesial and distal root fragments are broken 3 mm under the cervix on the mesial side and 7 mm under on the distal side. A longitudinal groove is still visible on the external aspect of the mesial root. In the inferior view, the pulp canals are filled with sediment.

NG0802.3 is a lower right second molar crown (LRM2) with a small root fragment (Figure 2j & Figure S10). The ivory enamel exhibits a slight hypoplasia 2 mm above the cervical margin. In occlusal view, the crown is sub-squared and is nearly unworn (stage 1). Four cusps are present according to the sequence Prd ≥ End>Hyd>Med. The Mmr is thick, high and complete, closing a small Fm with the moderately thick and incised MTC. The Dmr is thick and incised. A deep X groove pattern is visible. In lateral perspective, the mesial and distal faces are convex and vertical, while the buccal and lingual aspects are convex and sloping cervically. A large, ovoid and flat ICF is located in the buccal half of the mesial aspect, while the distal ICF, smaller, circular and flat, is in the middle of the distal aspect. The dark root fragment is broken just under the crown on the lingual side and 3 mm under on the buccal aspect. In the inferior view, the pulp cavity is visible, exhibiting four horns corresponding to each main cusp.

PCG09_KII_Z:1.37 represents a lower left second molar crown (LLM2) without roots (Figure 2k & Figure S11). The enamel, ivory to gray, shows a marked hypoplasia at the cervical level on the mesial, distal and buccal faces. The crown is sub-squared in occlusal view and nearly unworn (stage 1), with elevated reliefs and only small apical wear facets. Four cusps are present following the sequence End ≥Prd>Med>Hyd. The grooves are deep and display a clear X pattern. The Mmr and the Dmr are thick, elevated and incised. The MTC, moderately developed, low and interrupted, encloses a small and elongated Fm. In the lateral view, the mesial, distal and lingual aspects are convex and vertical, while the buccal aspect is flat and slopes cervically. A moderately developed, ovoid and flat ICF is located in the middle of the occlusal half of the mesial face. The distal ICF present the same characteristics despite being smaller. In inferior view, the pulp cavity is partially obstructed by sediment.

NG92.3 is a lower left second or third molar crown (LLM2/3) with a short root fragment (Figure 2l & Figure S12). The ivory enamel is well preserved and free of structural defects. The crown is ovoid in occlusal outline and moderately worn (stage 2) with slightly smoothed mesial cusps. Nonetheless, the occlusal distobuccal quarter exhibits an unusual large, but shallow MD oriented furrow. Five cusps display the pattern End>Med >Prd>Hyd>Hld. A+groove pattern is present. The Mmr, of moderate size, is slightly incised by the central groove and delimits a narrow Fm. A thick and interrupted MTC links the mesial cusps and the potential expression of a DTC is unclear. In lateral view, the distal, buccal and lingual aspects are convex, while the mesial one is flatter and nearly vertical. A small, ovoid and flat ICF is visible in the occlusobuccal part of the mesial aspect while no distal ICF is present. The black root fragment is broken just under the cervix on the lingual side and expands to 5 mm on the buccal aspect. In the inferior view, a mesial and a distal pulp canal are shown, the former being filled with sediment.

NG92 D6 ZE 57s/d 76 is a lower right second or third molar crown (LRM2/3), only preserving a small root fragment. The light-brown colored enamel is perfectly preserved (Figure 2m & Figure S13). Enamel hypoplasia is present all around the cervix. The specimen, square in occlusal view, is virtually unworn (stage 1) with small wear facets only at the cuspal apex. Four main cusps are well developed following the sequence Prd>End>Hyd>Med. The occlusal grooves are deep, especially in the center, and show an X pattern. The high and thin Mmr and Dmr are incised by the central groove. A high and thick disrupted MTC encloses the shallow and BL elongated Fm. The Fc is small but deep while the Fd is small and shallow, without clear boundaries. The occlusal surface is free of wrinkling. In lateral view, the mesial and distal aspects are convex and slightly diverging occlusally, while the buccal and lingual faces are convex and sloping cervically. A moderately developed, flat and ovoid ICF is located on the occluso-buccal part of the mesial face, while no distal ICF is visible. The roots are broken ca. 2 mm under the crown. In inferior view, the pulp cavity and its four pulp horns corresponding to each cusp are clearly discernible.

NG0802.2 is a lower right second or third molar crown (LRM2/3) with a small root fragment (Figure 2n & Figure S14). The ivory enamel exhibits no structural defect. The crown, showing a square outline in the occlusal view, is nearly unworn (stage 1) with only a slight apical smoothing. Six cusps are present and follow the sequence Prd>Med>End>Hld>Hyd>C6. A deep X groove pattern is expressed. The Mmr is complete, thick and moderately elevated. The Fm is small, enclosed by a thick incised MTC. The Fd is not well delimited mesially, while the Dmr is moderately thick and incised. In lateral view, the mesial face is convex and tilts occlusally, while the distal, buccal and lingual aspects are convex and vertical. A small, ovoid and flat ICF is located in the middle of the mesial aspect, while there is no distal ICF. The dark root fragment shows an irregular fracture, just under the crown on the lingual side and 4 mm under on the buccal side. In the inferior view, the pulp cavity is visible, showing four pulp horns corresponding to the four largest cusps.

NG9107.2 is a lower left third molar (LLM3), with an ivory crown and a light brown eroded and etched root fragment, preserved 7 mm under the cervix (Figure 2o & Figure S15). In occlusal view, the crown shows an ovo-rectangular outline. A slight enamel hypoplasia runs on the cervical margins of the mesial and lingual faces. Occlusal wear is moderate (stage 3) with relatively flattened and smoothed cusps and small brown points on the buccal cusps suggesting that dentine horns are underlying. Five main cusps are present: End >Prd>Med>Hld ≥ Hyd. A Y groove pattern is still discernible. The Mmr and Dmr were erased, as were the Fm and Fd. The Fc still retains some traces of a thick and interrupted MTC. Traces of wrinkling are still detectable. In lateral view, all faces are relatively flat and vertical. An elongated and slightly concave ICF occupies the center of the superior part of the mesial face, but no distal ICF is visible. The roots are strongly etched (possibly because of a digestive process) but still exhibit three root branches: a mesiobuccal, a mesiolingual and a distal. In inferior view, three pulp canals are visible, with the distal one being filled with sediment.

### Comparative Analyses

As the newly described sample mostly comprises isolated lower molars, a comparison with mandibular remains bearing *in situ* molars was preliminarily run to tentatively discriminate their serial position. The two specimens MI92.1 and NG92.2 exhibit a crown conformation which is similar to the first molars of NG8503, Sangiran 1b, Sangiran 22 and Sb8103, with a sub-pentagonal occlusal outline and a low MD extension [2], [12]. Also, the two crowns NG92.1 and NG92.4 resemble the second molar cusp arrangement and square occlusal outline of Sangiran 1b and Sangiran 22, while NG9107.2 is definitely analogous to Sangiran 1b, Sangiran 21 and Sangiran 22 third molar morphology, with a distal decrease of the talonid BL diameter. Nonetheless, because of the absence of distal ICF and of their small dimensions, the serial position of the three lower molars NG92.3, NG92 D6 ZE 57s/d 76 and NG0802.2 is still uncertain as second or third lower molars. However, two morphological traits, the reduction/absence of the Hld and the high frequency of the X and+groove patterns, distinguish the present Sangiran sample from most of the Lower-Middle Pleistocene available dental record (Table 2). While the Hld is absent in 50% of the new assemblage, this cusp is always well expressed in the second and third molar crowns of *H. habilis/rudolfensis* (HHR), African and Eurasian *H. erectus* (HEA, HEG, HEJ), the robust hominid specimens from Java (RHJ), *H. antecessor* (HA) and North African *H. heidelbergensis* (HHNA), but not in Chinese *H. erectus* third molars, which exhibit a tendency towards the loss of the fifth cusp (see ZKD B2-64 and F1−25 [88]). On the other hand, the four-cusped pattern is relatively frequent in European *H. heidelbergensis* (HHE), Neanderthals (NEA) and extant humans (EH) [89]−[91]. Concerning the occlusal groove pattern, here the X and+configurations represent the more frequent morphology (Table 2), whereas most of the previously described lower molars from the Kabuh Formation are reported to exhibit a Y groove pattern [8], , even if the+pattern is found in two *H. erectus* second molars (Sangiran 7−64 and 7−65 [8]) and in the third molar of the robust specimen Sangiran 9 [12]. It is noteworthy that the *Dryopithecus* groove configuration prevails in both second and third molars of specimens belonging to *H. habilis/rudolfensis*, African and Indonesian *H. erectus*, as well as in the second molars of *H. antecessor*, Georgian and Chinese *H. erectus.* Accordingly, a shift apparently occurred towards the Middle Pleistocene leading to an increasing frequency of the+pattern. Despite being already noticeable in the second molars at Dmanisi, its frequency increases in the third molars of the Zhoukoudian sample and in both second and third molars of North African and European *H. heidelbergensis*. In Neanderthals and extant humans, its occurrence is slightly less frequent. Finally, the X pattern is observed in European *H. heidelbergensis*, Neanderthals and extant human second and third molars, while Chinese *H. erectus* and *H. antecessor* tend to exhibit this pattern mostly in their third molars (Table 2).

**Table 2:** Proportions (% absence) of the Hld and occlusal groove pattern occurrence in the new Javanese fossil human dental sample compared to the figures from eleven extant and fossil human samples.

	N	Hld absence		groove pattern	
			Y	X	+
original sample	10	50%	20.0%	50.0%	30.0%
*comparative sample LM2s*					
HHR^a^	4	0.0%	100.0%	0.0%	0.0%
HEA^a^	5	0.0%	80.0%	0.0%	20.0%
HEG^a^	2	0.0%	50.0%	0.0%	50.0%
HA^a^	2	0.0%	100.0%	0.0%	0.0%
RHJb,c	3	0.0%	100.0%	0.0%	0.0%
HEJb,c,d	7	0.0%	71.4%	0.0%	28.6%
HEC^e^	5	0.0%	80.0%	0.0%	20.0%
HHNA^a^	3	0.0%	50.0%	0.0%	50.0%
HHE^a^	28	21.4%	42.3%	15.4%	42.3%
NEA^a^	29	17.2%	67.9%	17.9%	14.3%
EH^a^	136	81.6%	27.1%	35.3%	37.6%
*comparative sample LM3s*					
HHR^a^	3	0.0%	66.7%	0.0%	33.0%
HEA^a^	1	0.0%	100.0%	0.0%	0.0%
HEG^a^	1	0.0%	100.0%	0.0%	0.0%
HA^a^	1	0.0%	0.0%	100.0%	0.0%
RHJ^c^	1	0.0%	0.0%	0.0%	100.0%
HEJb,c,d	5	0.0%	100.0%	0.0%	0.0%
HEC^e^	4	50.0%	0.0%	25.0%	75.0%
HHNA^a^	4	0.0%	33.3%	0.0%	66.7%
HHE^a^	26	23.1%	23.8%	28.6%	52.4%
NEA^a^	23	30.4%	54.5%	31.8%	13.6%
EH^a^	81	55.0%	21.0%	53.2%	25.9%

a [90],

b [8],

c [12],

d [2],

e [88].

With regards to crown size, the lower molars included in the present new sample show relatively reduced dimensions. Their MD and BL diameters, computed crown area (CCA) and crown index (CI) have been compared here to the record from thirteen extant and Pleistocene human specimens/samples (Table 3).

**Table 3:** Linear (MD and BL in mm), surface (CCA in mm^2^) and proportion estimates (CI in %) of ten lower molar crowns from the new Javanese fossil human dental sample compared to the figures from thirteen extant and fossil human specimens/samples.

	*LM1*					*LM2*					*LM3*				
	n	MD	BL	CCA	CI	n	MD	BL	CCA	CI	n	MD	BL	CCA	CI
MI92.1	1	12.0	11.6	139.2	96.7										
NG92.2	1	11.2	10.8	121.0	96.4										
NG92.1						1	10.5	10.2	107.1	97.1					
NG92.4						1	10.1	9.5	96.0	94.1					
NG92.3						1	11.8	11.0	129.8	93.2	1	11.8	11.0	129.8	93.2
NG92.D6 ZE 57s/d 76						1	11.0	10.8	118.8	98.2	1	11.0	10.8	118.8	98.2
NG0802.2						1	11.3	10.7	120.9	94.7	1	11.3	10.7	120.9	94.7
NG0802.3						1	10.8	10.1	109.1	93.5	1				
PCG09_KII_Z1.37						1	10.1	10.0	101.0	99.0	1				
NG9107.2											1	10.9	10.1	110.1	92.6
HHR	11	13.8 (0.8)	12.1 (0.9)	167.8 (20.8)	87.9 (5.0)	12	15.3 (1.4)	13.5 (1.1)	207.6 (32.9)	88.4 (5.0)	11	15.6 (0.7)	13.3 (1.0)	208.7 (23.9)	85.4 (3.2)
HEA	8	12.7 (0.7)	11.2 (0.7)	143.0 (16.9)	88.0 (2.8)	7	13.3 (1.0)	12.0 (0.6)	160.3 (20.0)	90.7 (2.9)	7	14.2 (0.8)	12.2 (0.3)	174.0 (13.4)	86.2 (4.1)
HEG	4	13.1 (0.1)	11.9 (0.7)	156.0 (10.6)	90.8 (4.6)	6	13.0 (0.7)	11.8 (1.0)	153.7 (19.5)	90.3 (6.3)	4	12.4 (1.9)	11.8 (1.4)	146.3 (34.4)	95.9 (13.5)
HA	2	11.4	11.4	130.0	100.0	2	12.9	11.5	148.4	89.1	1	9.2	8.8	81.0	95.7
RHJ	1	13.0	13.0	169.0	100.0	4	14.5 (0.4)	13.5 (0.7)	195.7 (9.7)	93.5 (6.4)	2	14.0	13.6	189.9	96.7
HEJ	12	12.9 (0.8)	12.4 (0.8)	160.9 (19.1)	96.0 (4.6)	11	13.1 (0.7)	12.3 (1.0)	161.0 (19.5)	93.5 (4.0)	5	13.2 (1.5)	12.0 (0.8	159.7 (27.5)	91.4 (6.1)
HEC	12	12.6 (1.1)	11.8 (0.9)	149.2 (22.6)	94.1 (4.4)	8	12.3 (0.6)	11.8 (0.6)	145.8 (10.2)	96.1 (5.9)	9	11.5 (1.1)	11.2 (0.9)	129.4 (21.7)	97.6 (5.5)
HHNA	3	13.2 (0.9)	12.7 (0.3)	166.9 (14.2)	96.4 (4.6)	3	13.1 (0.9)	13.0 (0.7)	170.2 (19.5)	99.1 (2.6)	3	12.6 (0.7)	12.1 (0.5)	153.1 (14.2)	95.9 (2.3)
HHE	7	11.1 (0.4)	10.3 (0.5)	113.7 (9.2)	92.6 (2.6)	8	10.9 (0.9)	10.1 (0.9)	110.6 (19.3)	92.5 (2.9)	5	11.2 (1.2)	10.1 (0.9)	114.0 (19.8)	90.3 (5.3)
NEA	7	11.2 (0.5)	10.6 (0.7)	118.7 (12.0)	95.3 (3.7)	11	11.7 (0.5)	11.2 (0.6)	131.1 (11.3)	96.4 (4.1)	12	11.9 (0.6)	11.4 (0.6)	133.8 (11.6)	94.6 (4.4)
HF	2	10.1	10.7	101.0	99.0	2	10.2	10.2	104.0	100.1		8.7	9.5	82.7	91.6
FMH	79	11.5 (0.7)	10.9 (0.7)	124.7 (14.2)	95.3 (5.3)	80	11.0 (0.9)	10.7 (0.8)	118.4 (16.6)	97.6 (6.8)	54	10.8 (1.1)	10.7 (1.0)	116.1 (19.9)	100.2 (10.8)
EH	174	11.4 (0.6)	11.0 (0.5)	124.9 (11.0)	96.3 (4.2)	204	10.7 (0.6)	10.5 (0.6)	113.4 (11.7)	98.4 (4.2)	185	10.6 (0.7)	10.4 (0.7)	110.3 (13.5)	98.2 (5.1)

The estimates of three indeterminate specimens (NG92.3, NG92.D6 ZE 57s/d 76, NG0802.2) are duplicated into the LM2 and LM3 columns.

**Figure 3:**
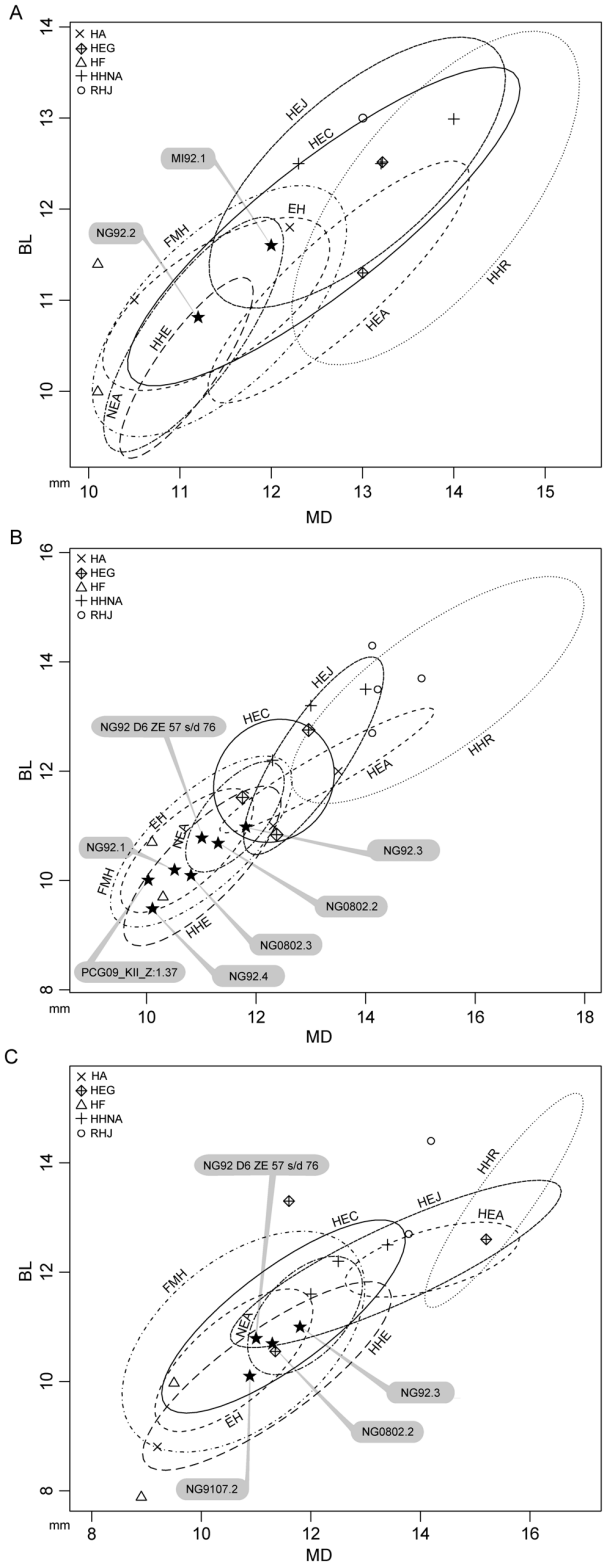
MD vs. BL diameter plots for the LM1 (A), LM2 (B) and LM3 crowns (C) from the new Javanese fossil human dental sample compared to the figures from thirteen extant and fossil human samples represented by 95% confident equiprobable ellipses. The specimens NG92.3, NG92.D6 ZE 57s/d 76 and NG0802.2 for which the serial attribution is uncertain appear in both LM2 and LM3 graphs.

The first molars MI92.1 and NG92.2 exhibit relatively small MD, BL and CCA values, being in the lower range of the Lower-Middle Pleistocene Asian series, including Javanese (HEJ) and Chinese (HEC) *H. erectus* (Figure 3A). On the other hand, as indicated by the proportionally inverse CI (96.7% and 96.4%, respectively), their modest MD elongation shows the highest affinities with Javanese *H. erectus* (HEJ; 96.0%), North African *H. heidelbergensis* (HHNA; 96.4%) and extant humans (EH, 96.3%).

The four second molars NG92.1, NG92.4, NG0802.3 and PCG09_KII_Z:1.37, as well as the indeterminate second or third molars NG92 D6 ZE 57s/d 76 and NG0802.2, also show relatively modest dimensions (in average, MD: 10.5 mm, BL: 10.2 mm, CCA: 108.8 mm^2^), outside the known range of most Lower and early Middle Pleistocene specimens, including Indonesian and Chinese *H. erectus* (Figure 3B). Conversely, their estimates are encompassed by European *H. heidelbergensis* (HHE), fossil modern (FMH) and extant (EH) human distributions, and also fall close to the Flores hominins (HF). However, the specimen NG92.3 falls within the lower variation range of both Chinese (HEC) and Javanese *H. erectus* (HEJ). With this regard, it has to be noted that the Javanese robust hominid specimens (RHJ), kept apart from the Indonesian sample because of their unique morphological and dimensional patterns [12]−[13], mostly exceed the range of classic *H. erectus* (HEJ), and this despite a shared MD extension (CI: 93.5%). Besides, the CI of the four second molars and of the three second or third molars (on average, 95.7%) fit most of the MD estimates available for Chinese *H. erectus* (96.1%) and Neanderthals (NEA; 96.4%).

The third molar NG9107.2 (MD: 10.9 mm, BL: 10.1 mm, CCA: 110.1 mm^2^) exhibits lower dimensions compared to the Indonesian fossil samples considered in this study, but fits the variation range globally expressed by Chinese *H. erectus*, European *H. heidelbergensis*, fossil and extant modern humans (Figure 3C). The three uncertain second or third molar crowns fall in the range exhibited by the Javanese *H. erectus* lower third molar (Figure 3C). In addition, for their MD, BL and CCA values, they are compatible with the variation ranges displayed by the Chinese *H. erectus* and European *H. heidelbergensis* samples, and fall close to the late Upper Pleistocene LB1 specimen from Flores. Besides, the newly reported Sangiran assemblage expresses an elevated CI (ranging from 92.6% to 98.2%), thus indicating a lower MD elongation of the crown than commonly observed in *H. habilis/rudolfensis* (85.4%), African (86.2%) and Javanese *H. erectus* (91.4%), European *H. heidelbergensis* (90.3%) and *H. floresiensis* (91.6%).

**Figure 4:**
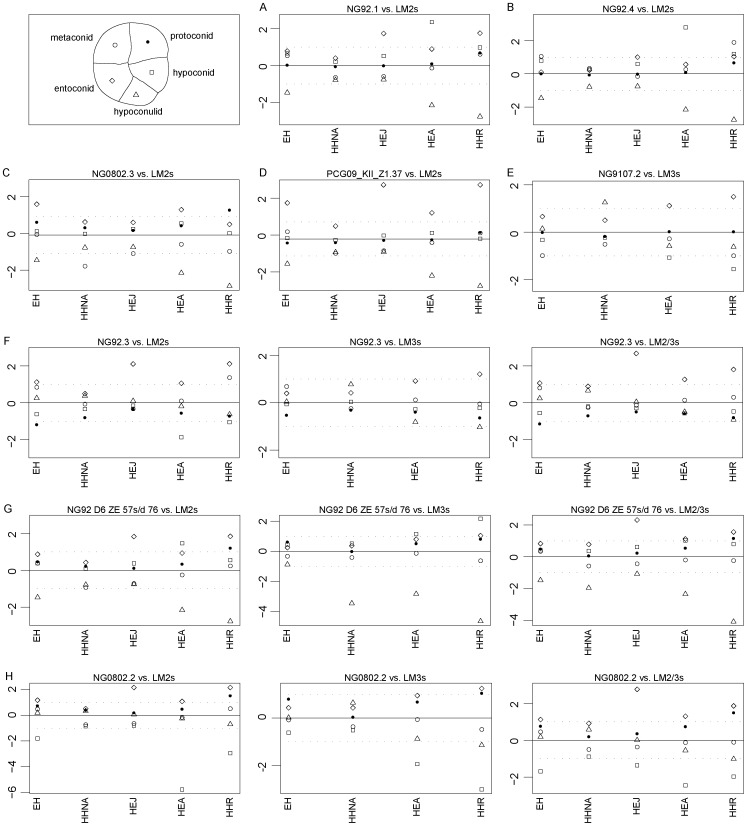
Adjusted Z-score graphs of relative molar cusp area in NG92.1 (A), NG92.4 (B), NG0802.3 (C), PCG09_KII_Z1.37 (D), NG9107.2 (E), NG92.3 (F), NG92.D6 ZE 57s/d 76 (G), and NG0802.2 (H) from the new Javanese fossil human dental sample compared to the LM2 and/or LM3 figures from five extant and fossil human samples. The full line passing through the zero represents the mean and the dotted lines correspond to the estimated 95% limit of variation expressed for each group. Each symbol corresponds to a distinct cusp base area for the tested specimen.

Finally, the relative cusp areas of selected second and third molars from the present Javanese sample were compared to six extant and Pleistocene human groups (Table 4, Figure 4 and Table S2).

**Table 4:** Cusp area estimates of eight LM2 and LM3 crowns from the new Javanese fossil human dental sample compared to the figures from six extant and fossil human specimens/samples.

	n	Prd	Med	Hyd	End	Hld
*original sample LM2s*						
NG92.1	1	27.5	22.9	25.9	23.7	0.0
NG92.4	1	27.4	25.3	26.9	20.4	0.0
NG0802.3	1	30.6	20.1	21.8	27.5	0.0
PCG09_KII_Z1.37	1	26.1	22.4	21.4	30.1	0.0
*original sample LM2/3s*						
NG92.3	1	20.9	24.3	16.4	25.3	13.1
NG92.D6 ZE 57s/d 76	1	29.9	22.2	23.9	24.1	0.0
NG0802.2	1	36.0	21.5	17.3	19.8	5.5
*original sample LM3*						
NG9107.2	1	25.0	20.8	12.2	27.4	14.6
*comparative sample LM2s*						
HHR^a^	7	24.3 (1.8)	21.7 (0.7)	21.3 (1.8)	15.6 (1.7)	17.0 (2.3)
HEA^a^	3	26.6 (3.3)	23.7 (2.0)	20.6 (0.7)	14.7 (3.3)	14.4 (2.2)
HA^b^	1	25.5	15.0	25.4	20.5	13.5
HEJ^c^	3	27.8 (3.9)	26.1 (1.1)	18.6 (2.8)	15.9 (0.9)	11.6 (3.1)
HHNA^c^	3	28.0 (1.7)	24.5 (0.5)	22.4 (3.5)	16.2 (3.7)	8.9 (2.3)
EHb,c	77	27.4 (2.6)	20.5 (2.3)	21.1 (3.7)	20.1 (2.6)	10.8 (4.2)
*comparative sample LM3s*						
HHR^a^	6	24.5 (2.5)	24.8 (1.7)	17.1 (1.1)	16.9 (2.7)	16.7 (1.4)
HEA^a^	5	25.5 (3.5)	22.4 (2.8)	18.0 (2.1)	15.1 (3.9)	19.1 (2.0)
HEJ^c^	1	25.1	22.6	19.4	17.1	15.8
HHNA^c^	3	30.5 (6.1)	27.5 (2.7)	15.8 (3.2)	15.5 (4.7)	10.7 (0.6)
EH^c^	5	25.1 (2.6)	22.9 (0.7)	17.3 (5.1)	22.2 (2.6)	12.5 (4.7)

Into parentheses, the s.d.

a [75],

b [84],

c original data.

NG92.1, NG92.4 and PCG09_KII_Z:1.37 are included in the 95% confidence interval of the cusp area variation displayed by North African *H. heidelbergensis* (Figure 4A, B, D), NG92.4 also fitting the cuspal proportions more typical of Javanese *H. erectus* lower second molars (Figure 4B). The cusp pattern of NG0802.3 corresponds to the condition expressed by Javanese *H. erectus*, but not to that shown by any other Pleistocene or extant group included in these analyses (Figure 4C). Similarly, the third molar NG9107.2, while approaching the extant human condition, does not resemble any of the fossil samples represented in this study (Figure 4E). The three molars for which the serial position is ambiguous (Figure 4F–H) show a high statistical degree of similarity with the penecontemporaneous North African *H. heidelbergensis* second molar. However, as third molar, NG92.3 would be compatible with African *H. erectus*, the Algerian sample and some extant humans. Moreover, NG0802.2 fits the North African *H. heidelbergensis* and extant human patterns, whereas NG92 D6 ZE 57s/d 76 only corresponds to the latter.

Following these analyses, the three specimens NG92.3, NG92 D6 ZE 57s/d 76 and NG0802.2 shows no significant features for an unambiguous serial identification and can thus represent either a second or a third molar.

## Discussion

Around the Lower-Middle Pleistocene boundary, environmental changes occurred in Southeast Asia [92]. They primarily resulted from global climate modifications associated to changes in orbital forcing leading to higher amplitude alternations of glacial/interglacial climates and to higher eustatic variations (rev. in [93]). A strengthening of the East Asian monsoon related to a slowing down of the thermohaline oceanic circulation and some intermittent accelerations of the Himalayan tectonic uplifts are also evoked among the factors having contributed to dramatically parcel out the region [92], . As a consequence, pronounced latitudinal shifts in vegetation and faunas occurred, mainly between North Chinese colder open lands and the tropical *Stegodon*-*Ailuropoda* faunal complex area covering the South mainland and insular Southeast Asia [96]−[97]. Particularly in the Indonesian archipelago, this ecological fragmentation led to highly endemic fauna with few predators, thus favoring the development of local evolutionary morphs [96]−[98]. Within this framework, the dentognathic human fossil record of Java has been recognized as one of the most morpho-dimensionally heterogeneous in the Lower-Middle Pleistocene (rev. in [13]). In fact, while most specimens are generally considered within the “typical” size range of *H. erectus s.s.* [29], [38], [39−41], , marked variation is expressed within this hypodigm, notably because of the extreme robusticity characterizing specimens such as Sangiran 4, Sangiran 5 or Sangiran 6a [12]−[13]. Currently available evidence suggests that, together with the Dmanisi fossil assemblage [99], the morpho-dimensional features displayed by early African *H. ergaster* and the Javanese *H. erectus* dentognathic specimens from the Pucangan/Grenzbank levels are similar and only slightly derived with respect to the ancestral *Homo* condition, providing support to the frequent lumping of African and Asian subsamples for broad evolutionary studies [45]. In this context, the dentognathic remains from the Lower Pleistocene Pucangan Formation and the unconformably overlying “Grenzbank Zone” generally exhibit some relatively primitive features and, more importantly, greater robusticity compared to the Kabuh Formation-derived specimens [12−13], . This, with the notable exceptions of Sangiran 1b and 22, which show a relatively gracile mandibular corpus despite their absolutely large teeth.

Compared to the estimates on the earlier dental sample from the Kabuh Formation [12−13], , the newly reported assemblage shows distinct post-canine structural reduction [12−13], , an evidence which could cast doubts about its taxonomic allocation to *H. erectus*. However, for example, the second molar PCG09_KII_Z:1.37, discovered *in situ* during excavations of the late Lower-early Middle Pleistocene Kabuh Formation outcropping at the Pucung site, is among the smallest specimens, and other molars show dimensional values well compatible with the variation range of Chinese *H. erectus* and European *H. heidelbergensis*. Also, when the cusp base areas are especially considered, most of the crowns from the new assemblage fit the figures displayed by the Lower-Middle Pleistocene dental samples from Java (*H. erectus*) and Algeria (*H. heidelbergensis*) included in the comparative analyses. Only the third molar NG9107.2 and, whenever considered as third molars, the three specimens for which the serial position is uncertain, more closely approximate the extant human pattern. However, two among these specimens (NG92.3 and NG0802.2) also distinctly fit the evidence from the early Middle Pleistocene remains from Tighenif.

In sum, because of their original provenance context, preservation conditions and degree of mineralisation shared with other Lower-Middle Pleistocene specimens from Sangiran, strong intra-sample morpho-dimensional homogeneity and, mostly, because of their substantial affinities with the dental record of Chinese *H. erectus* and, to a lesser extent, African *H. heidelbergensis*, we preliminarily assign the present set of fifteen permanent isolated tooth crowns from the Kabuh Formation of Sangiran to *H. erectus s.s*.

The new sample from the Sangiran Dome provides additional evidence of significant morpho-dimensional crown variation and time-related structural reduction characterizing the Indonesian human dental assemblage from the Lower to the Middle Pleistocene [12−13], . Besides post-canine crown size decrease, additional changes throughout the Middle Pleistocene observed in Javanese *H. erectus* craniodental features include general facial reduction to a greater extent than observed in late African *H. ergaster*, dental arcade widening, lowering of the mandibular ramus [12−13], [45], . Similar morphostructural trends have been also reported for the Middle Pleistocene Chinese samples from Zhoukoudian and Lantian [41], [88], [100], which reveal an even more advanced degree of third molar reduction [12−13], [45], [91], . In this framework, the establishment of some regionally-specific morphological variants is traceable at macro-regional scale, notably between the Chinese and Javanese groups [18], [45]. The morphological features of the Chinese tooth sample also recall the penecontemporaneous European pattern, pointing towards frequent longitudinal interactions occurred across Eurasia during the early Middle Pleistocene [18], [91].

An evolutionary scenario implying intermittent isolation of the Javanese groups from the Northern continental demes is also suggested by a number of differences in cranial morphology, such as a larger biasterionic breadth, a more massive and vertical supraorbital torus, a more convex infraorbital region observed in the Indonesian hypodigm [45]. However, rather than resulting from a series of local microevolutive events occurred within the same population, recent evidence support the view that the changes observed during the transition from the Lower Pleistocene dentognathic morphology represented by the Pucangan assemblage from Sangiran to the derived condition exhibited by the Kabuh specimens more likely resulted either from replacement or introgression by admixture with continental immigrating groups [12−13], [18], .

### Conclusions

The comparative analysis of the newly reported sample of fifteen isolated permanent tooth crowns from the Kabuh Formation of the Sangiran Dome, here preliminarily allocated to the *H. erectus s.s*. hypodigm, has provided additional evidence of significant morpho-dimensional variation and time-related structural reduction characterizing the Indonesian human fossil assemblage notably during the transition from the Lower to the Middle Pleistocene [12−13], . With respect to the estimates available for the earlier dentognathic material from the Pucangan Formation and the “Grenzbank Zone”, globally exhibiting more primitive features and greater robusticity, the molars from the Kabuh Formation distinctly show lower mesiodistal crown extension associated with marked reduction or even absence of the hypoconulid, and low frequency of the Y pattern. The combination of these characteristics, rare in the Lower and early Middle Pleistocene human dental record, notably in the early specimens from Sangiran [12]−[13], is more commonly found in Chinese *H. erectus* and, to a minor extent, in North African *H. heidelbergensis*, an evidence which highlights the complex nature of the intermittent exchanges occurred between continental and insular Southeast Asia, as well as of the longitudinal evolutionary dynamics across continental Asia around the Lower to Middle Pleistocene boundary.

## Supporting Information

Figure S1 **The specimen MI92.2.** B, buccal; D, distal; I, inferior; L, lingual; M, mesial; O, occlusal. Scale bar is 1 cm.
(TIF)

Figure S2 **The specimen NG9505.** B, buccal; D, distal; I, inferior; L, lingual; M, mesial; O, occlusal. Scale bar is 1 cm.
(TIF)

Figure S3 **The specimen NG91-G10 n°1.** B, buccal; D, distal; I, inferior; L, lingual; M, mesial; O, occlusal. Scale bar is 1 cm.
(TIF)

Figure S4 **The specimen PDS0712.** B, buccal; D, distal; I, inferior; L, lingual; M, mesial; O, occlusal. Scale bar is 1 cm.
(TIF)

Figure S5 **The specimen NG0802.1.** B, buccal; D, distal; I, inferior; L, lingual; M, mesial; O, occlusal. Scale bar is 1 cm.
(TIF)

Figure S6 **The specimen MI92.1.** B, buccal; D, distal; I, inferior; L, lingual; M, mesial; O, occlusal. Scale bar is 1 cm.
(TIF)

Figure S7 **The specimen NG92.2.** B, buccal; D, distal; I, inferior; L, lingual; M, mesial; O, occlusal. Scale bar is 1 cm.
(TIF)

Figure S8 **The specimen NG92.1**. B, buccal; D, distal; I, inferior; L, lingual; M, mesial; O, occlusal. Scale bar is 1 cm.
(TIF)

Figure S9 **The specimen NG92.4.** B, buccal; D, distal; I, inferior; L, lingual; M, mesial; O, occlusal. Scale bar is 1 cm.
(TIF)

Figure S10 **The specimen NG0802.3.** B, buccal; D, distal; I, inferior; L, lingual; M, mesial; O, occlusal. Scale bar is 1 cm.
(TIF)

Figure S11 **The specimen PCG09_KII_Z:1.37.** B, buccal; D, distal; I, inferior; L, lingual; M, mesial; O, occlusal. Scale bar is 1 cm.
(TIF)

Figure S12 **The specimenNG92.3.** B, buccal; D, distal; I, inferior; L, lingual; M, mesial; O, occlusal. Scale bar is 1 cm.
(TIF)

Figure S13 **The specimen NG92 D6 ZE 57 s/d 76.** B, buccal; D, distal; I, inferior; L, lingual; M, mesial; O, occlusal. Scale bar is 1 cm.
(TIF)

Figure S14 **The specimenNG0802.2.** B, buccal; D, distal; I, inferior; L, lingual; M, mesial; O, occlusal. Scale bar is 1 cm.
(TIF)

Figure S15 **The specimen NG9107.2.** B, buccal; D, distal; I, inferior; L, lingual; M, mesial; O, occlusal. Scale bar is 1 cm.
(TIF)

Table S1 **The comparative dental record used for the assessment of crown size, Hld development, occlusal groove pattern, and cusp proportions.**
(DOC)

Table S2 **The comparative dental record used for the assessment of relative cusp areas (in %).**
(DOCX)

## References

[1] Dubois E (1891) Palaeontologische onderzoekingen op Java. Versl Mijnw Batavia 3: 12–14.

[2] von Koenigswald GHR (1940) Neue *Pithecanthropus*-Funde 1936−1938. Wet Meded Dienst Mijnb Ned Oost-Indië 28: 1–223.

[3] von Koenigswald GHR (1967) Evolutionary trends in the deciduous molars of the hominidea. J Dent Res 46: 777–786. 10.1177/00220345670460053001 5234017

[4] Jacob T (1973) Paleoanthropological discoveries in Indonesia with special reference to the finds of the last two decades. J Hum Evol 2: 473–485.

[5] Grine FE (1984) Comparison of the deciduous dentition of African and Asian hominids. Cour Forsch-Inst Senckenberg 69: 69–82.

[6] Widianto H (1991) The hominid dental remains of Java: A metrical study. Bull Indo-Pac Prehist Assoc 11: 23–35.

[7] Widianto H (1993) Unité et diversité des hominidés fossiles de Java : présentation de restes humains inédits. PhD dissertation. Paris: MNHN.

[8] Grine FE, Franzen JL (1994) Fossil hominid teeth from the Sangiran dome (Java, Indonesia). Courier Forsch Senckenberg 171: 75–103.

[9] Aziz F, Saefudin I (1996) An isolated tooth of orang-utan (*Pongo pygmaeus*) from the Sangiran area, Central Java, Indonesia. Geol Res Dev Centre Bandung 8: 47–50.

[10] Aziz F (2001) Hominid fossils housed at the Geological Research and Development Center, Bandung, Indonesia. In: Indriati E, editor. A scientific life: papers in honor of Prof. Dr. T. Jacob. Yogyakarta: Bigraf Publishing. 53–66.

[11] Kaifu Y, Aziz F, Baba H (2001) New evidence for the existence of *Pongo* in the Early/Middle Pleistocene Java. In: Sudijono, Aziz F, editors. Towards ahead: Geological Museum in a Changing World. Papers presented in the International Symposium on Geological Museum. August 22–24, 2000. Bandung, Indonesia. Geological Research and Development Centre Special Publication 27. Bandung: Geological Research and Development Centre. 55–60.

[12] Kaifu Y, Aziz F, Baba H (2005a) Hominid mandibular remains from Sangiran: 1952–1986 collection. Am J Phys Anthropol 128: 497–519. 15761881 10.1002/ajpa.10427

[13] Kaifu Y, Baba H, Aziz F, Indriati E, Schrenk F, et al (2005b) Taxonomic affinities and evolutionary history of the Early Pleistocene hominids of Java: dentognathic evidence. Am J Phys Anthropol 128: 709–726. 15761880 10.1002/ajpa.10425

[14] Kaifu Y, Arif J, Yokoyama K, Baba H, Suparka E, et al (2007) A new *Homo erectus* molar from Sangiran. J Hum Evol 52: 222–226. 17123585 10.1016/j.jhevol.2006.08.012

[15] Indriati E (2004) Indonesian fossil hominid discoveries from 1889–2003: catalogue and problems. Nat Sci Mus Monographs 24: 163–177.

[16] Aziz F, Kaifu Y, Baba H (2006) A new mandibular molar of *Pongo* from Sangiran, Central Java. In: Zaim Y, Rizal Y, Aswan, Fitriana BS, editors. S. Sartono: dari Hominid ke delapsi dengan kontroversi. Bandung: LIPI Press. 69–72.

[17] Arif J, Kapid R, Kaifu Y, Baba H, Abdurrahman M (2007) Announcement of GLOM 2006.03: a four isolated deciduous teeth from Sangiran, Central Java, Indonesia. In: Indriati E, editor. Recent advances on southeast Asian paleoanthropology and archaeology. Yogyakarta: Gadjah Mada University. 140–150.

[18] Zaim Y, Ciochon RL, Polanski JM, Grine FE, Bettis EA III, et al (2011) New 1.5 million-year-old *Homo erectus* maxilla from Sangiran (Central Java, Indonesia). J Hum Evol 61: 363–376. 21783226 10.1016/j.jhevol.2011.04.009

[19] Zanolli C (2011) The endostructural organization of the late Lower-early Middle Pleistocene human dental remains from Indonesia and Africa, with a special attention to *Homo erectus s.s*. Comparative high-resolution characterization and taxonomic problems. PhD dissertation. Paris: Muséum national d’Histoire naturelle.

[20] Zanolli C, Bondioli L, Mancini L, Mazurier A, Widianto H, et al (2012) Two human fossil deciduous molars from the Sangiran Dome (Java, Indonesia): outer and inner morphology. Am J Phys Anthropol 147: 472–481. 22281866 10.1002/ajpa.21657

[21] Weidenreich F (1945) Giant early Man from Java and South China. Anthropol Pap Am Mus 40: 1–134. 10.1126/science.99.2581.479 17792233

[22] von Koenigswald GHR (1950) Fossil hominids from the Lower Pleistocene of Java. 18th Intl Geol Cong 9: 59–61.

[23] von Koenigswald GHR (1954) *Pithecanthropus*, *Meganthropus* and the Australopithecinae. Nature 173: 795–797. 13165651 10.1038/173795a0

[24] von Koenigswald GHR (1960) *Meganthropus palaeojavanicus* v.K. a new fossil hominid from Java. Cong Intl Anthropol Ethnol. Brussels: 271–272.

[25] Robinson JT (1953) *Meganthropus*, Australopithecines and Hominids. Am J Phys Anthropol 11: 1–28. 13040502 10.1002/ajpa.1330110112

[26] Robinson JT (1955) Further remarks on the relationship between *Meganthropus* and australopithecines. Am J Phys Anthropol 13: 429–445. 13275577 10.1002/ajpa.1330130304

[27] Sartono S (1961) Notes on a new find of a *Pithecanthropus* mandible. Publikasi Teknik Seri Paleont 2: 1–51.

[28] Sartono S (1980) Pre-sapiens migration in Southeast Asia. In: Intl Assoc Historians Asia VIIIth Conf Kualalumpur (Malaysia), August 1980.

[29] Le Gros Clark WE (1964) The Fossil Evidence for Human Evolution, 2nd ed. Chicago: University of Chicago Press.

[30] Tobias PV, von Koenigswald GHR (1964) A comparison between the Olduvai hominines and those of Java, and some implications for hominid phylogeny. Nature 204: 515–518. 14238152 10.1038/204515a0

[31] Jacob T (1980) The *Pithecanthropus* of Indonesia: phenotype, genetics and ecology. In: Königsson LK, editor. Current argument on early man. Oxford: Pergamon Press. 170–179.

[32] Krantz GS (1975) An explanation for the diastema of Javan *erectus* skull IV. In: Tuttle RH, editor. Paleoanthropology, Morphology and Paleoecology. La Hague: Mouton. 361–372.

[33] Krantz GS (1981) The process of human evolution. Cambridge: Schenkman Publishing Company.

[34] Krantz GS (1994) The palate of skull Sangiran 4 from Java. Cour Forsch Inst Senckenberg 171: 69–74.

[35] Orban-Segebarth R, Procureur F (1983) Tooth size of *Meganthropus palaeojavanicus*: an analysis of distances between some fossil hominids and a modern human population. J Hum Evol 12: 711–720.

[36] Franzen JL (1985a) Asian australopithecines? In: Tobias PV, editor. Hominid Evolution: Past, Present, and Future. New York: Wiley-Liss. 255–263.

[37] Franzen JL (1985b) What is “*Pithecanthropus dubius* Koenigswald, 1950”? In: Delson E, editor. Ancestors: the hard evidence. New York: Alan R Liss. 221–226.

[38] Kramer A (1989) The Evolutionary and Taxonomic Affinities of the Sangiran Mandibles of Central Java, Indonesia. PhD dissertation. Ann Arbor: University of Michigan.

[39] Rightmire GP (1993) The evolution of *Homo erectus*. New York: Cambridge University Press.

[40] Kramer A, Konigsberg LW (1994) The phyletic position of Sangiran 6 as determined by multivariate analysis. Courier Forsch Senckenberg 171: 105–114.

[41] Wolpoff MH (1999) Paleoanthropology, 2nd ed. Boston: McGraw-Hill.

[42] Tyler DE (2001) *Meganthropus*: cranial fossils from Java. Hum Evol 16: 81–101.

[43] Tyler DE (2003) Sangiran 5, (“*Pithecanthropus dubius*”), *Homo erectus*, “*Meganthropus*”, or *Pongo* ? Hum Evol 18: 229–242.

[44] Tyler DE (2004) An examination of the taxonomic status of the fragmentary mandible Sangiran 5, (*Pithecanthropus dubius*), *Homo erectus*, “*Meganthropus*”. or *Pongo*? Quat Intl 117: 125–130.

[45] Antón SC (2003) Natural history of *Homo erectus* . Yearb Phys Anthopol 46: 126–170. 10.1002/ajpa.10399 14666536

[46] Schwartz JH, Tattersall I (2003) The human fossil record. Craniodental morphology of genus *Homo* (Africa and Asia), vol. 2. Hoboken: Wiley–Liss.

[47] Djubiantono T, Sémah F (1991) Lower Pleistocene marine-continental transitional beds in the Solo depression and their relation to the environment of the Pucangan hominids. Bull Indo-Pac Prehist Assoc 11: 7–13.

[48] Sémah F, Sémah AM, Djubiantono T, Simanjuntak HT (1992) Did they also make stone tools ? J Hum Evol 23: 439–446.

[49] Saleki H (1997) Apport d’une intercomparaison des méthodes nucléaires (230Th/234U, ESR et 40 Ar/39Ar) à la datation de couches fossilifères pléistocènes dans le dôme de Sangiran (Java; Indonésie). PhD dissertation. Paris: MNHN.

[50] Sémah F (2001) La position stratigraphique du site de Ngebung 2 (Dôme de Sangiran, Java Central, Indonesie). In: Sémah AM, Sémah F, Falguéres C, Grimaud-Hervé D, editors. Origine des Peuplements et Chronologie des Cultures Paléolithiques dans le Sud-Est Asiatique. Paris: Artcom’. 299–330.

[51] Sémah AM (1998) Pollen analysis and the palaeoenvironmental evolution of the Solo depression with special reference to the Sangiran dome. In: Simanjuntak T, Prasetyo B, Handini R, editors. Sangiran: Man, Culture, and Environment in Pleistocene Times, Proceedings of the International Colloquium on Sangiran Solo – Indonesia. Jakarta: The National Research Centre of Archaeology/Ecole Francaise d’Extreme-Orient/Yayasan Obor Indonesia. 231–256.

[52] Sémah AM, Sémah F (2001) La signification paléoécologique des couches à hominidés de l′île de Java. In: Sémah AM, Sémah F, Falguéres C, Grimaud-Hervé D, editors. Origine des Peuplements et Chronologie des Cultures Paléolithiques dans le Sud-Est Asiatique. Paris: Artcom’. 251–278.

[53] Moigne AM, Sémah F, Sémah AM, Bouteaux A, Due Awe R (2004) Mammalian fossils from two sites of the Sangiran Dome (Central Jawa, Indonesia), in the biostratigraphical framework of the Jawanese Pleistocene. In: Maul LC, Kahlke RD, editors. Late Neogene and Quaternary biodiversity and evolution: Regional developments and interregional correlations. 18 th International Senckenberg Conference. VI International Palaeontological Colloquium in Weimar. Weimar (Germany), 25th –30th April, 2004. Terra Nostra, Schriften der Alfred-Wegener-Stiftung 2. Stuttgart: Schweizerbart’sche Verlagsbuchhandlung. 176–178.

[54] Bouteaux A, Moigne AM (2010) New taphonomical approaches: The Javanese Pleistocene open-air sites (Sangiran, central Java). Quat Intl 223–224: 220–225.

[55] Sémah AM, Sémah F, Djubiantono T, Brasseur B (2010) Landscapes and hominids’ environments: changes between the Lower and the Early Middle Pleistocene in Java (Indonesia). Quat Intl 223–224: 451–454.

[pone.0067233-Larick1] Larick R, Ciochon RL, Zaim Y, Sudijono Suminto, et al (2001) Early Pleistocene 40Ar/39Ar ages for Kabuh Formation hominins, Central Java, Indonesia. Proc Natl Acad Sci U S A 98: 4866–4871. 11309488 10.1073/pnas.081077298 PMC33129

[57] Bettis III EA, Milius AK, Carpenter SJ, Larick R, Zaim Y, et al (2009) Way out of Africa: Early Pleistocene paleoenvironments inhabited by *Homo erectus* in Sangiran, Java. J Hum Evol 56: 11–24. 19007966 10.1016/j.jhevol.2008.09.003

[58] Hyodo M, Watanabe N, Sunata W, Susanto EE, Wahyono H (1993) Magnetostratigraphy of hominid fossil bearing formations in Sangiran and Mojokerto, Java. Anthrop Sci 101: 157–186.

[59] Hyodo M (2001) The Sangiran geomagnetic excursion and its chronological contribution to the Quaternary geology in Java. In: Simanjuntak HT, Prasetyo B, Handini R, editors. Sangiran: man, culture and environment in Pleistocene times. Jakarta: Yayasan Obor Indonesia. 320–335.

[60] Sémah F, Saleki H, Falguères C, Féraud G, Djubiantono T (2000) Did Early Man reach Java during the Late Pleistocene? J Archaeol Sci 27: 763–769.

[61] Langbroek M, Roebroeks W (2000) Extraterrestrial evidence on the age of the hominids from Java. J Hum Evol 38: 595–600. 10715199 10.1006/jhev.1999.0394

[62] Antón SC, Swisher CC III (2004) Early dispersals of *Homo* from Africa. Annu Rev Anthropol 33: 271–296.

[63] Hyodo M, Matsu’ura S, Kamishima Y, Kondo M, Takeshita Y, et al (2011) High–resolution record of the Matuyama–Brunhes transition constrains the age of Javanese *Homo erectus* in the Sangiran dome, Indonesia. Proc Natl Acad Sci U S A 108: 19563–19568. 22106291 10.1073/pnas.1113106108 PMC3241771

[64] Djubiantono T (1992) Les derniers dépôts marins de la dépression de Solo (Java Central, Indonésie). Chronostratigraphie et paléogéographie. PhD dissertation. Paris: MNHN.

[65] Lee AYC, Jacob T, Deniaux B (2004) Preliminary examination of buccal dental microwear in Javanese hominids. Bull Indo–Pac Prehist Assoc 24: 143–152.

[66] Smith HB (1984) Patterns of molar wear in hunter–gatherers and agriculturalists. Am J Phys Anthropol 63: 39–56. 6422767 10.1002/ajpa.1330630107

[67] Turner CG II, Nichol CR, Scott GR (1991) Scoring procedures for key morphological traits of the permanent dentition: the Arizona State University Dental Anthropology System. In: Kelley M, Larsen C, editors. Advances in dental anthropology. New York: Wiley–Liss. 13–31.

[68] Scott GR, Turner CG II (1997) The anthropology of modern human teeth. Dental morphology and its variation in recent human populations. Cambridge: Cambridge University Press.

[69] Moorrees CFA, Thomsen SO, Jensen E, Yen PKJ (1957) Mesiodistal crown diameters of deciduous and permanent teeth. J Dent Res 36: 39–47. 13398501 10.1177/00220345570360011501

[70] Mounier A, Marchal F, Condemi S (2009) Is *Homo heidelbergensis* a distinct species? New insight on the Mauer mandible. J Hum Evol 56: 219–246. 19249816 10.1016/j.jhevol.2008.12.006

[71] Grine FE, Smith HF, Heesy CP, Smith EJ (2009) Phenetic affinities of Plio–Pleistocene *Homo* fossils from South Africa: molar cusp proportions. In: Grine FE, Fleagle JG, Leakey RE, editors. The first humans. Origin and early evolution of the genus *Homo*. New York: Springer. 49–62.

[72] Leakey MG, Spoor F, Dean MC, Feibel CS, Antón SC, et al (2012) New fossils from Koobi Fora in northern Kenya confirm taxonomic diversity in early *Homo* . Nature 488: 201–204. 22874966 10.1038/nature11322

[73] Voisin JL, Condemi S, Wolpoff MH, Frayer DW (2012) A new online database (http://anthropologicaldata.free.fr) and a short reflection about the productive use of compiling Internet data. PaleoAnthropol. 2012: 241–244.

[74] Wood BA, Abbott SA, Graham SH (1983) Analysis of the dental morphology of Plio–Pleistocene hominids. II. Mandibular molars – study of cusp areas, fissure pattern and cross sectional shape of the crown. J Anat 137: 287–314. 6415025 PMC1171822

[75] Wood BA (1991) Koobi Fora research project. Vol. 4. Hominid cranial remains from Koobi Fora. Oxford: Clarendon Press.

[76] Wood BA, Engleman CA (1988) Analysis of the dental morphology of Plio-Pleistocene hominids. V. Maxillary postcanine tooth morphology. J Anat 161: 1–35. 3254883 PMC1262088

[77] Suwa G, Wood BA, White TD (1994) Further analysis of mandibular molar crown and cusp areas in Pliocene and early Pleistocene hominids. Am J Phys Anthropol 93: 407–426. 8048464 10.1002/ajpa.1330930402

[78] Suwa G, White TD, Howell FC (1996) Mandibular postcanine dentition from the Shungura Formation, Ethiopia: crown morphology, taxonomic allocations, and Plio-Pleistocene hominid evolution. Am J Phys Anthropol 101: 247–282. 8893088 10.1002/(SICI)1096-8644(199610)101:2<247::AID-AJPA9>3.0.CO;2-Z

[79] Bailey SE (2004) A morphometric analysis of maxillary molar crowns of Middle-Late Pleistocene hominins. J Hum Evol 47: 183–198. 15337415 10.1016/j.jhevol.2004.07.001

[80] Bailey SE, Liu W (2010) A comparative dental metrical and morphological analysis of a Middle Pleistocene hominin maxilla from Chaoxian (Chaohu), China. Quat Intl 211: 14–23.

[81] Uchida A (1998a) Variation in tooth morphology of *Pongo pygmaeus* . J Hum Evol 34: 71–79. 9467782 10.1006/jhev.1997.0187

[82] Uchida A (1998b) Variation in tooth morphology of *Gorilla gorilla* . J Hum Evol 34: 55–70. 9467781 10.1006/jhev.1997.0186

[83] Uchida A (1998c) Design of the mandibular molar in the extant great apes and Miocene fossil hominoids. Anthropol Sci 106: 119–126.

[84] Bermúdez de Castro JM, Rosas A, Nicolás ME (1999) Dental remains from Atapuerca-TD6 (Gran Dolina site, Burgos, Spain). J Hum Evol 37: 523–566. 10496999 10.1006/jhev.1999.0323

[85] Maureille B, Rougier H, Houet F, Vandermeersch B (2001) Les dents inférieures du Néandertalien Regourdou 1 (site de Regourdou, commune de Montignac, Dordogne): analyses métriques et comparatives. Paleo 13: 183–200.

[86] Scolan H, Santos F, Tillier AM, Maureille B, Quintard A (2012) Des nouveaux vestiges néanderthaliens à Las Pélénos (Monsempron-Libos, Lot-et-Garonne, France). Bull Mém Soc Anthropol Paris 24: 69–95.

[87] Grimaud-Hervé D., Widianto H (2001) Les fossiles humains découverts à Java depuis les années 1980. In: Sémah F, Falguères C, Grimaud-Hervé D, Sémah AM, editors. Origine des peuplements et chronologie des cultures paléolithiques dans le Sud-est asiatique. Paris: Artcom’. 331–358.

[88] Weidenreich F (1937) The dentition of *Sinanthropus pekinensis*: a comparative odontography of the hominids. Palaeontol Sin Ser D 1: 1–180.

[89] Bailey SE (2000) Dental morphological affinities among late Pleistocene and recent humans. Dent Anthropol 14: 1–8.

[90] Martinón-Torres M (2006) Evolución del aparato dental en homínidos: estudio de los dientes humanos del Pleistoceno de Sierra de Atapuerca (Burgos). PhD dissertation. University of Santiago de Compostella: Santiago de Compostella.

[91] Martinón-Torres M, Bermúdez de Castro JM, Gómez-Robles A, Arsuaga JL, Carbonell E, et al (2007) Dental evidence on the hominin dispersals during the Pleistocene. Proc Natl Acad Sci U S A 104: 13279–13282. 17684093 10.1073/pnas.0706152104 PMC1948952

[92] Dennell RW (2004) Hominid dispersals and Asian biogeography during the Lower and early Middle Pleistocene, c. 2.0–0.5 Mya. Asian Persp 43: 205–226.

[93] Kingston JD (2007) Shifting adaptive landscapes: Progress and challenges in reconstructing early hominid environments. Yearb Phys Anthropol 50: 20–58. 10.1002/ajpa.20733 18046753

[94] Heinrich R, Baumann KH, Huber R, Meggers H (2002) Carbonate preservation records of the past 3 Myr in the Norwegian-Greenland Sea and the northern North Atlantic: implication for the history of NADW production. Mar Geol 184: 17–39.

[95] Liu Z, Trentesaux A, Clemens SC, Colin C, Wang P, et al (2003) Clay mineral assemblages in the northern South China Sea: implications for East Asian monsoon evolution over the past 2 million years. Mar Geol 201: 133–146.

[96] Ciochon RL (2009) The mystery ape of Pleistocene Asia. Nature 458: 910–911. 10.1038/459910a 19536242

[97] Ciochon RL (2010) Divorcing hominins from the Stegodon-Ailuropoda fauna: new views on the antiquity of hominins in Asia. In: Fleagle JG, Shea JJ, Grine FE, Leakey REF, editors. Out of Africa I: The first hominin colonization of Eurasia. New York: Springer. 111–126.

[98] Louys J, Turner A (2012) Environment, preferred habitats and potential refugia for Pleistocene *Homo* in Southeast Asia. C R Palevol 11: 203–211.

[99] Martinón-Torres M, Bermúdez de Castro JM, Gómez-Robles A, Margvelshvill A, Prado L, et al (2008) Dental remains from Dmanisi (Republic of Georgia): Morphological analysis and comparative study. J Hum Evol 55: 249–273. 18486183 10.1016/j.jhevol.2007.12.008

[100] Kaifu Y (2006) Advanced dental reduction in Javanese *Homo erectus* . Anthropol Sci 114: 35–43.

[101] Kaifu Y, Zaim Y, Baba H, Kurniawan I, Kubo D, et al (2011a) New reconstruction and morphological description of a *Homo erectus* cranium: Skull IX (Tjg-1993.05) from Sangiran, Central Java. J Hum Evol 61: 270–294. 21683428 10.1016/j.jhevol.2011.04.002

