# Supplementary figures and images for: Additional Evidence for Morpho-Dimensional Tooth Crown Variation in a New Indonesian H. erectus Sample from the Sangiran Dome (Central Java)
Source: PLoS One. 2013 Jul 3;8(7):e67233. doi: 10.1371/journal.pone.0067233 (PMC3700995; doi:10.1371/journal.pone.0067233)

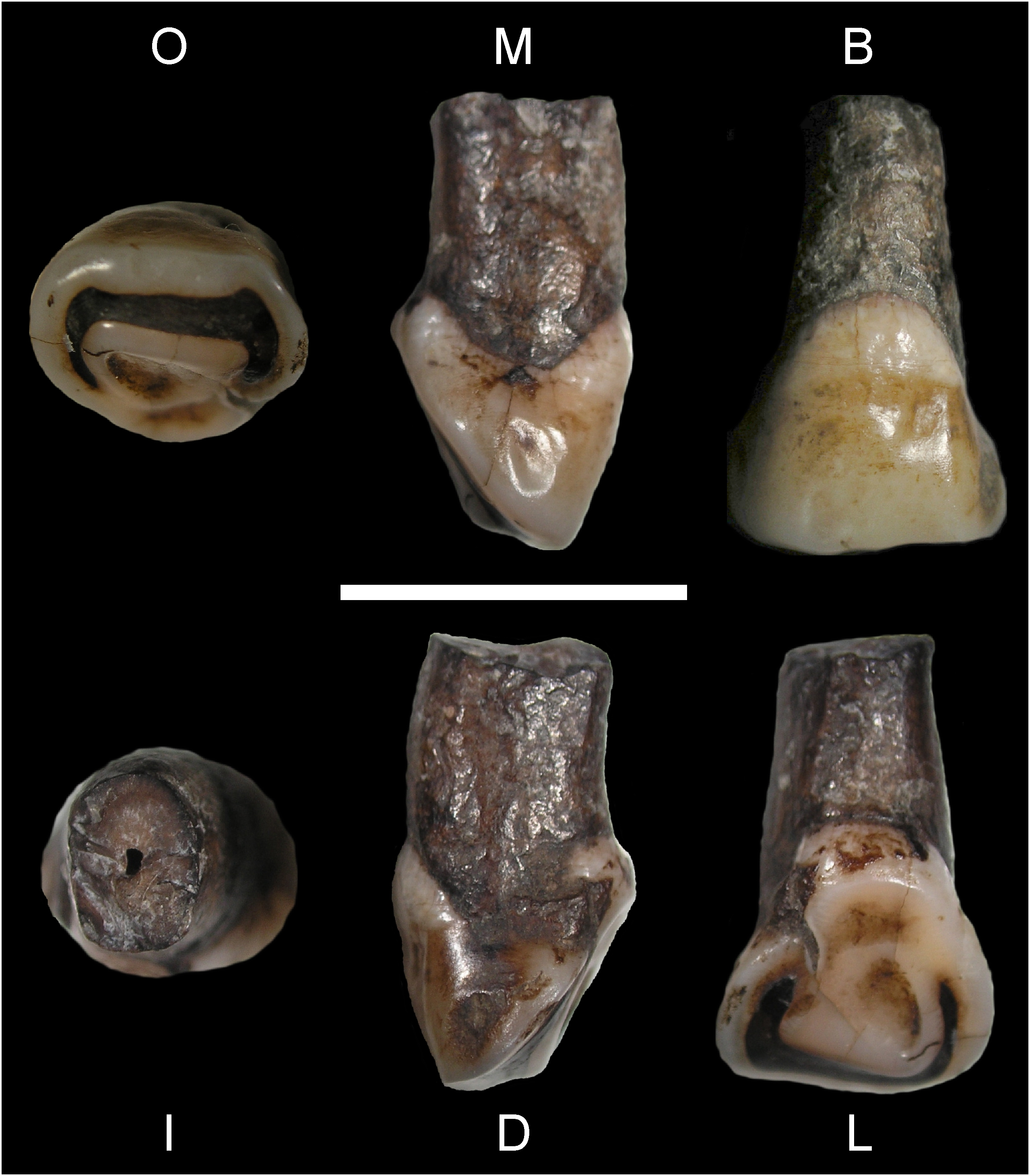

Supplement: Figure S1 — The specimen MI92.2. B, buccal; D, distal; I, inferior; L, lingual; M, mesial; O, occlusal. Scale bar is 1 cm. (TIF) [file pone.0067233.s001.tif]

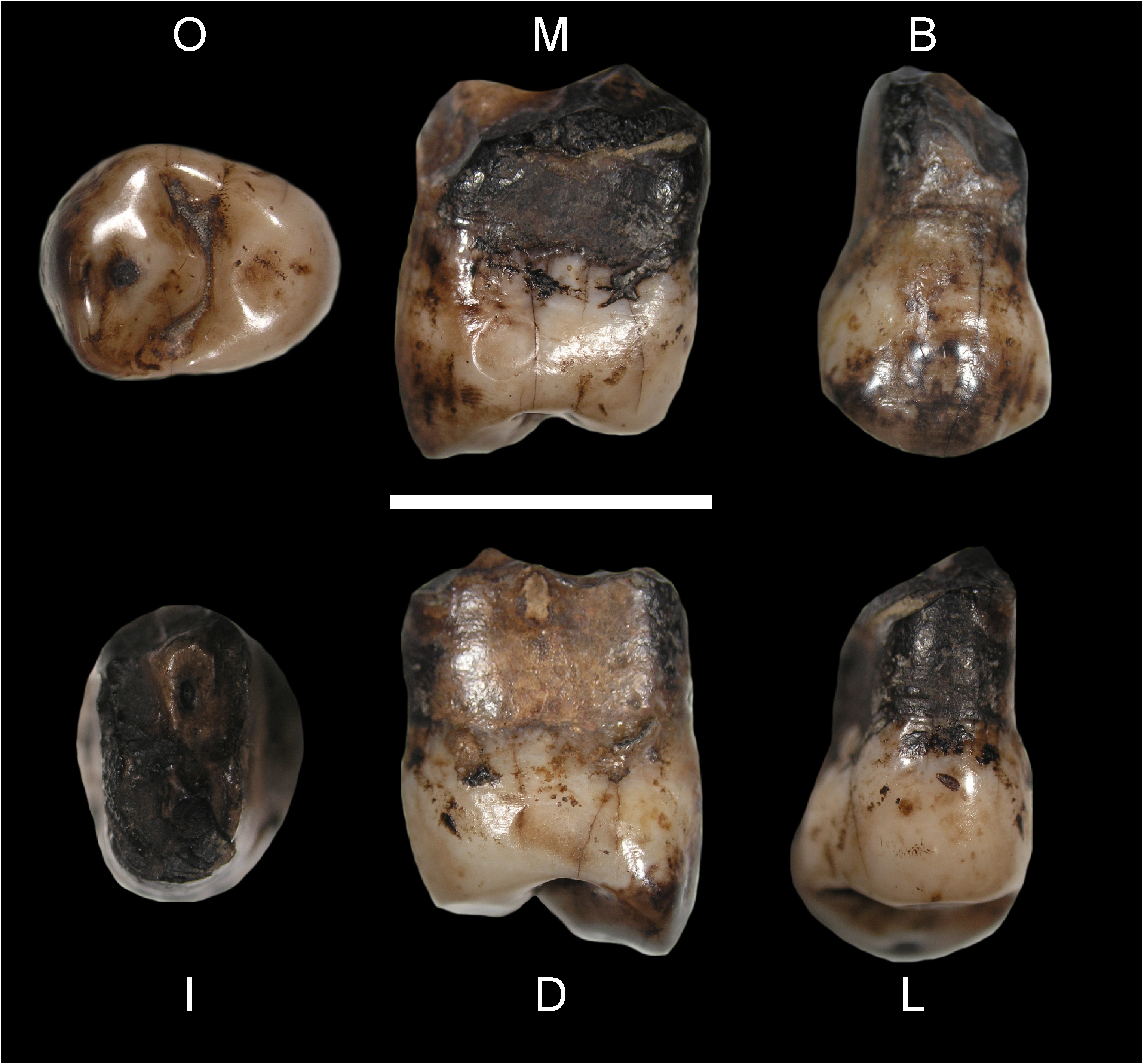

Supplement: Figure S2 — The specimen NG9505. B, buccal; D, distal; I, inferior; L, lingual; M, mesial; O, occlusal. Scale bar is 1 cm. (TIF) [file pone.0067233.s002.tif]

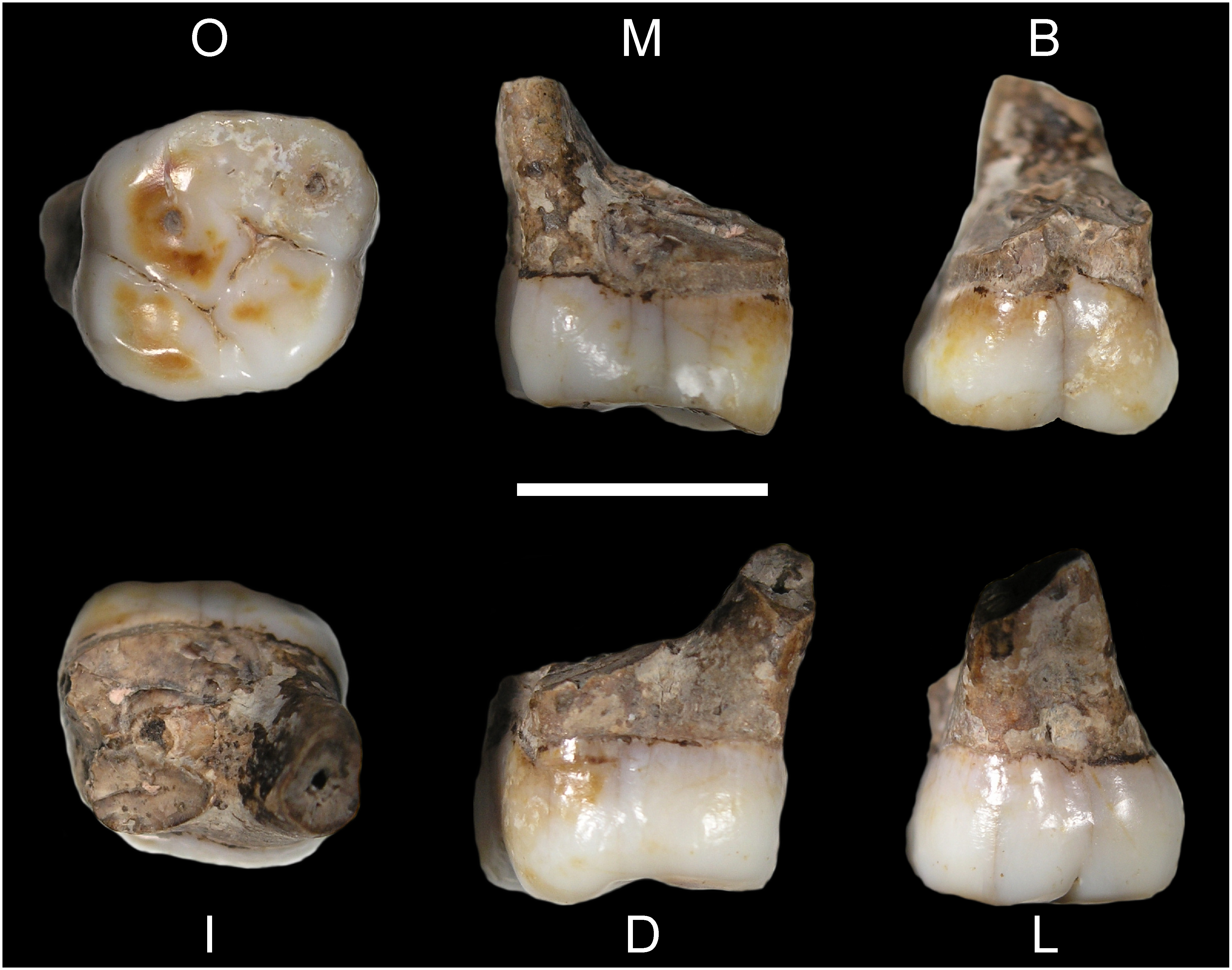

Supplement: Figure S3 — The specimen NG91-G10 n°1. B, buccal; D, distal; I, inferior; L, lingual; M, mesial; O, occlusal. Scale bar is 1 cm. (TIF) [file pone.0067233.s003.tif]

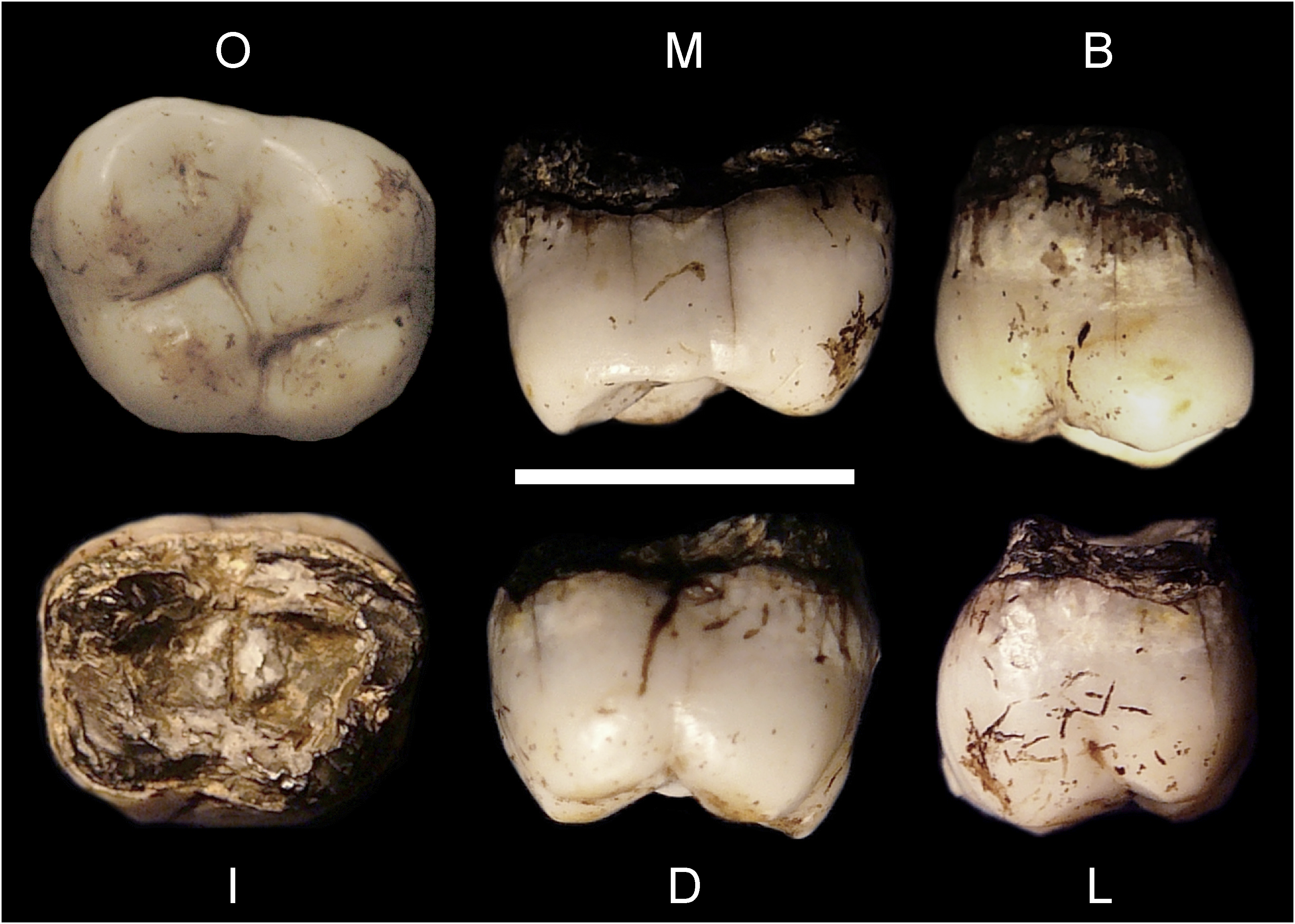

Supplement: Figure S4 — The specimen PDS0712. B, buccal; D, distal; I, inferior; L, lingual; M, mesial; O, occlusal. Scale bar is 1 cm. (TIF) [file pone.0067233.s004.tif]

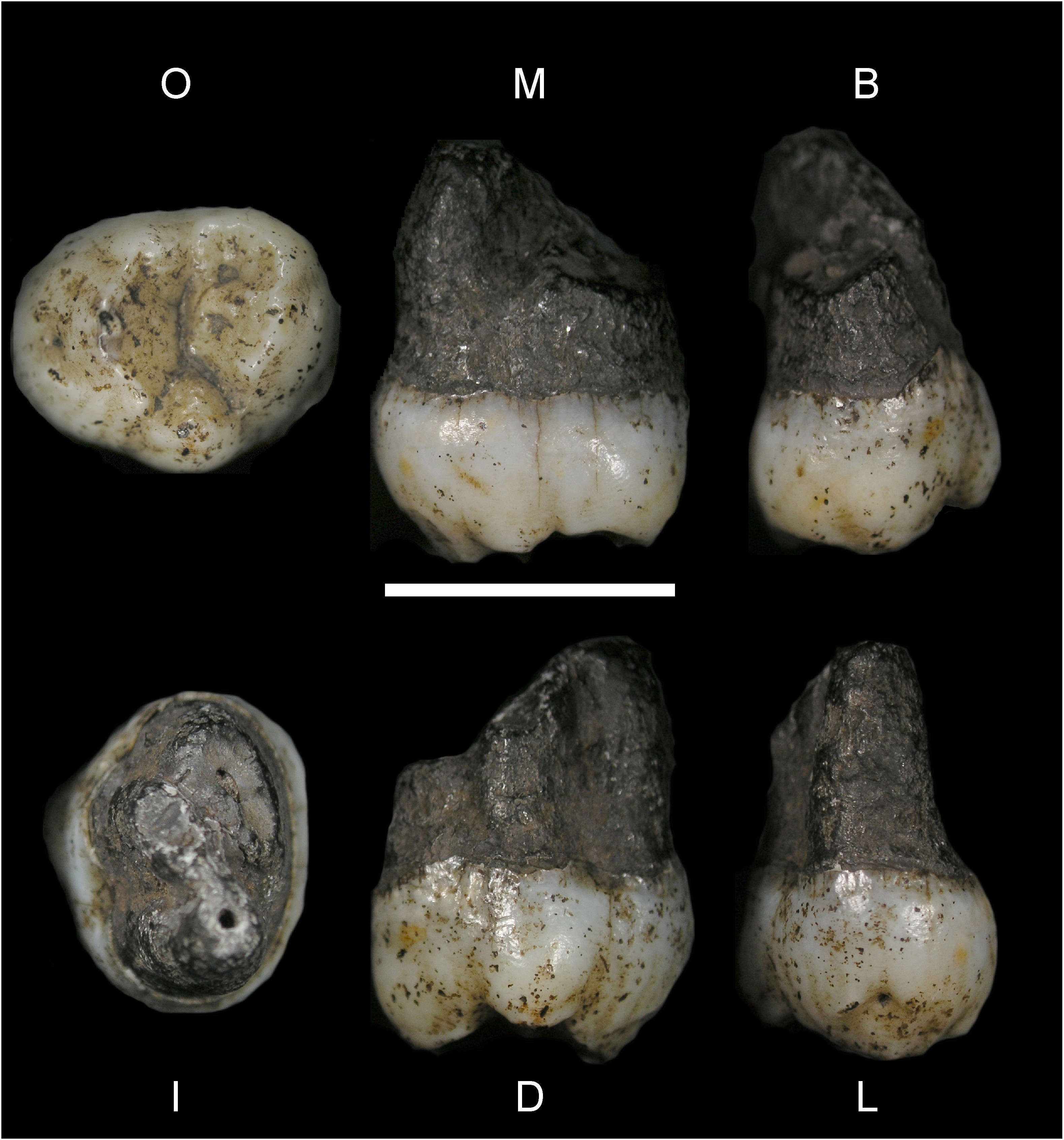

Supplement: Figure S5 — The specimen NG0802.1. B, buccal; D, distal; I, inferior; L, lingual; M, mesial; O, occlusal. Scale bar is 1 cm. (TIF) [file pone.0067233.s005.tif]

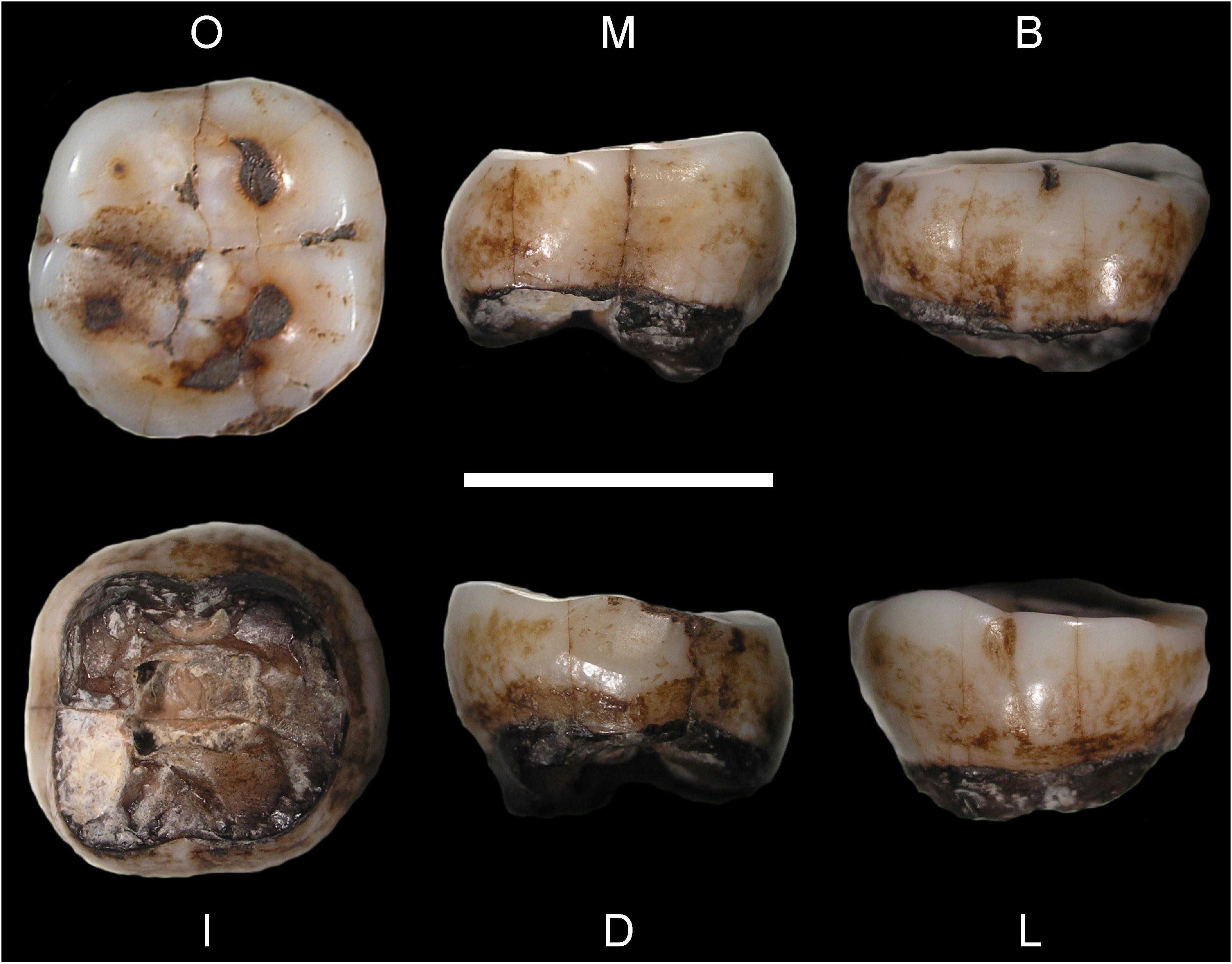

Supplement: Figure S6 — The specimen MI92.1. B, buccal; D, distal; I, inferior; L, lingual; M, mesial; O, occlusal. Scale bar is 1 cm. (TIF) [file pone.0067233.s006.tif]

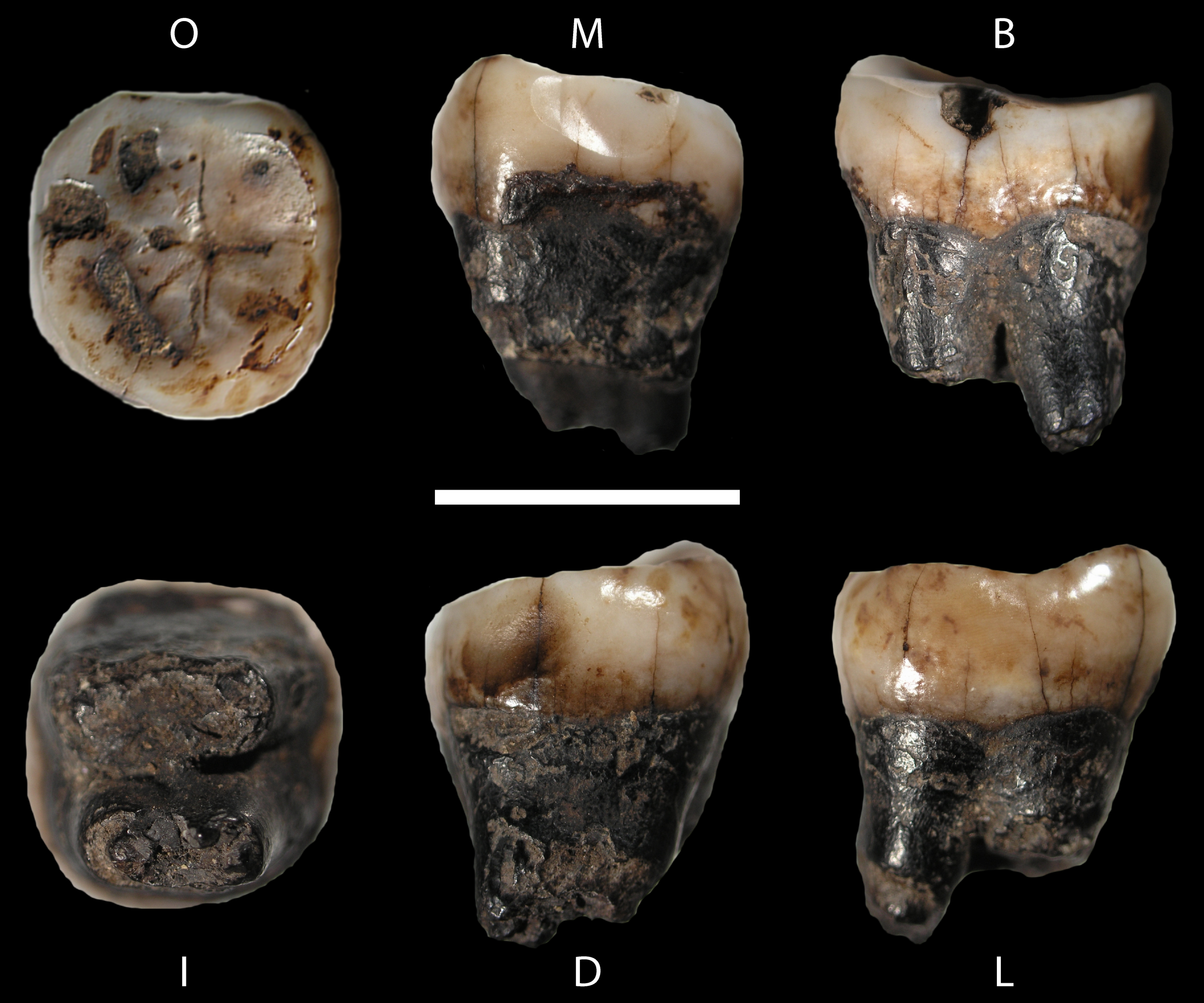

Supplement: Figure S7 — The specimen NG92.2. B, buccal; D, distal; I, inferior; L, lingual; M, mesial; O, occlusal. Scale bar is 1 cm. (TIF) [file pone.0067233.s007.tif]

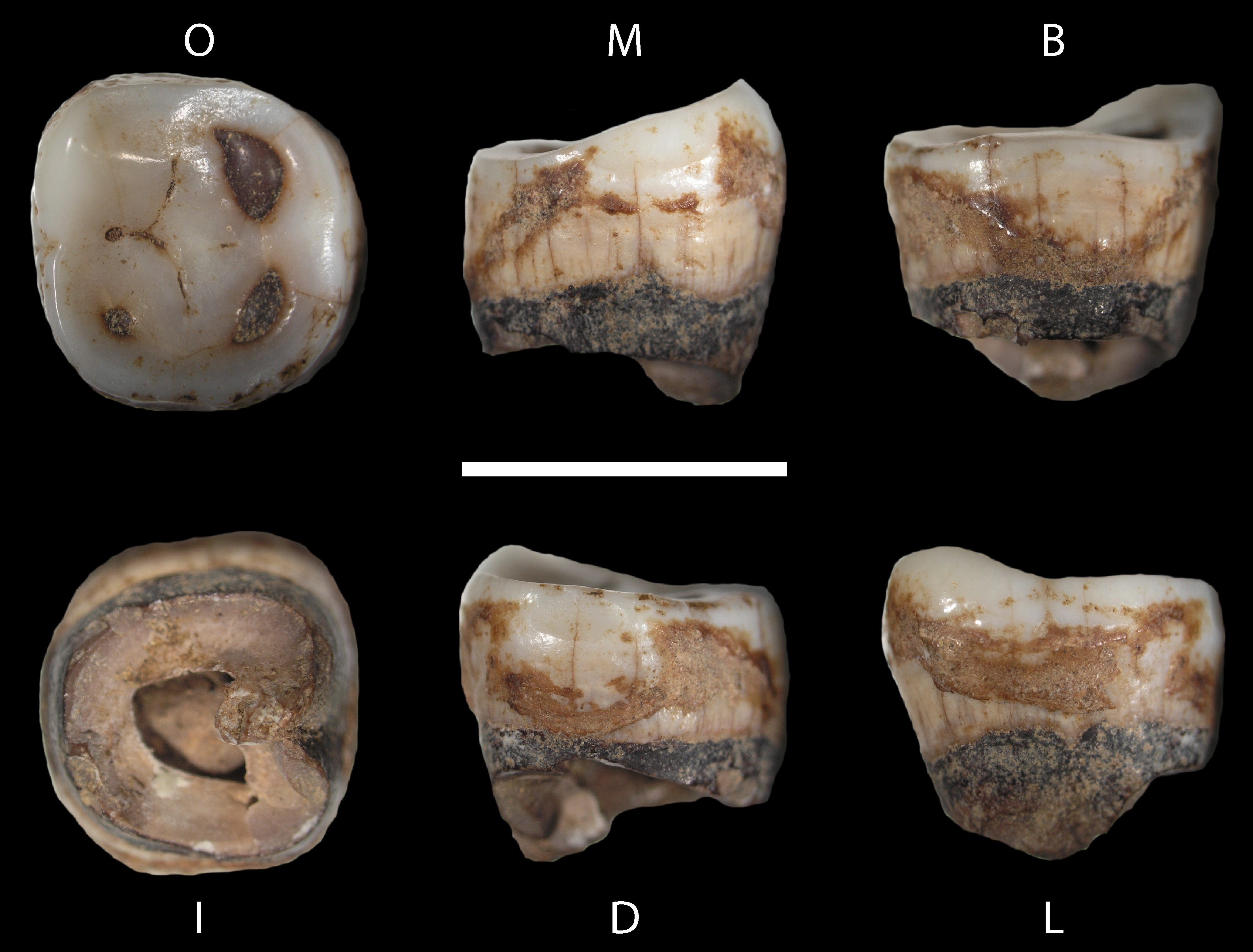

Supplement: Figure S8 — The specimen NG92.1. B, buccal; D, distal; I, inferior; L, lingual; M, mesial; O, occlusal. Scale bar is 1 cm. (TIF) [file pone.0067233.s008.tif]

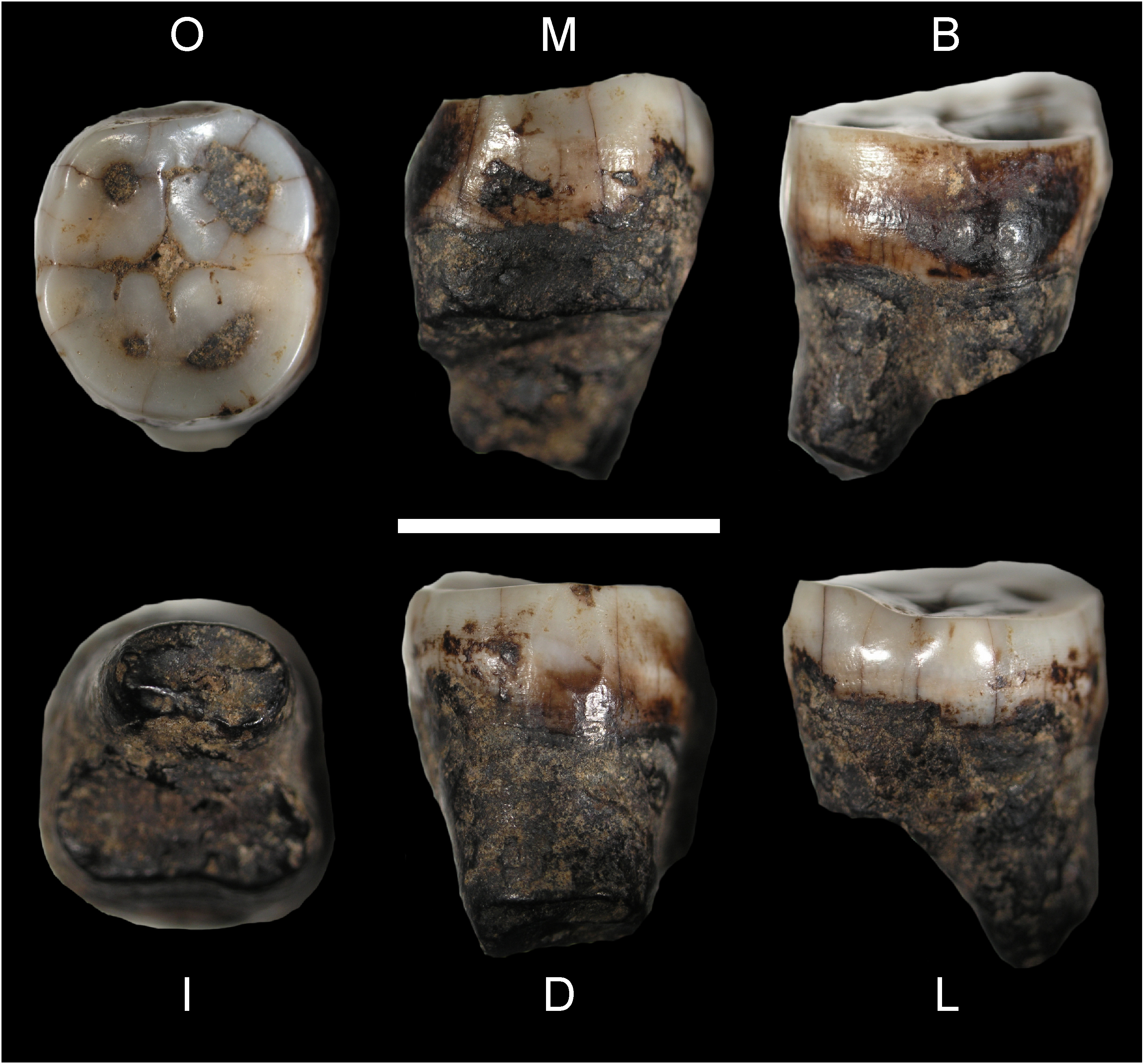

Supplement: Figure S9 — The specimen NG92.4. B, buccal; D, distal; I, inferior; L, lingual; M, mesial; O, occlusal. Scale bar is 1 cm. (TIF) [file pone.0067233.s009.tif]

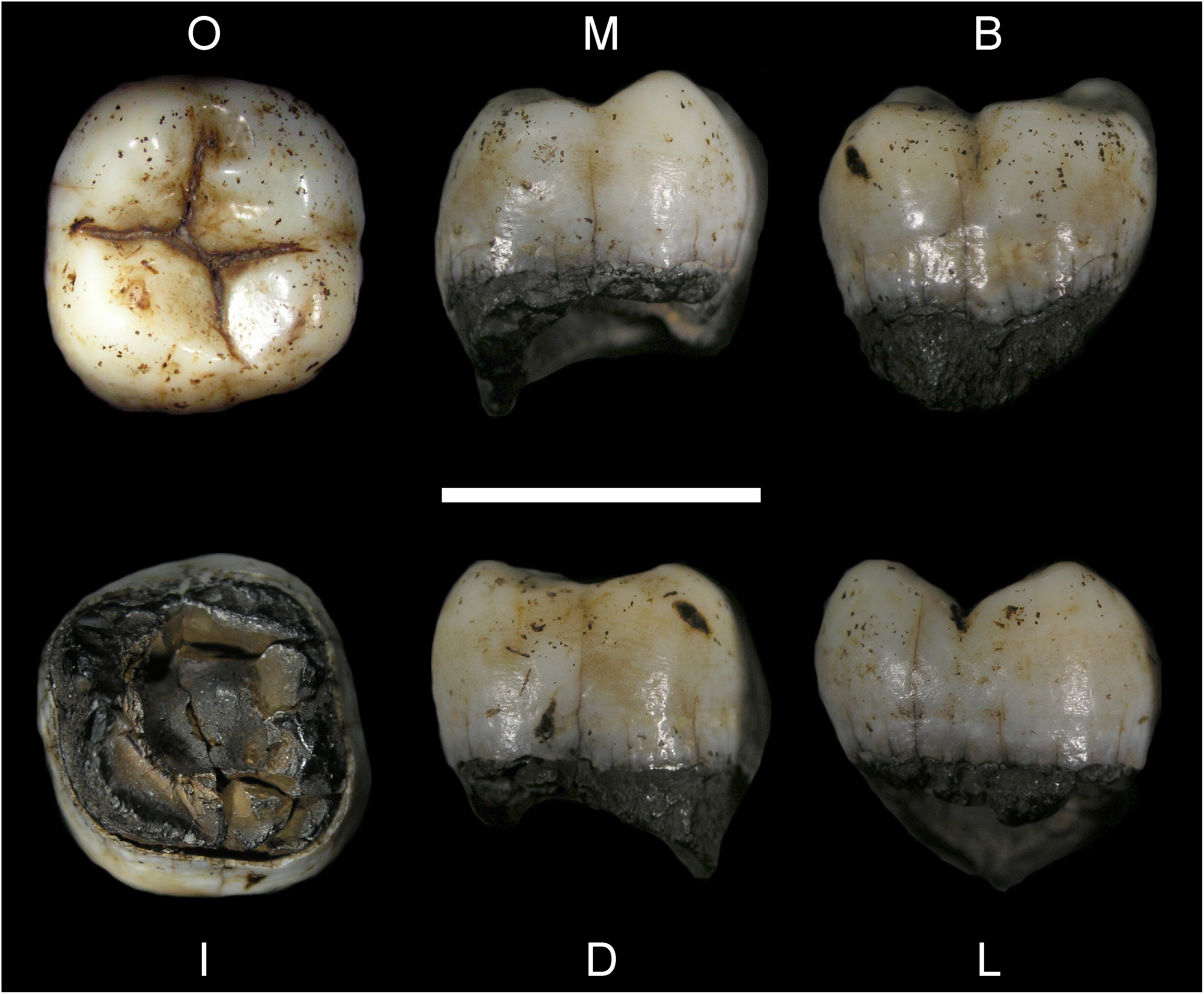

Supplement: Figure S10 — The specimen NG0802.3. B, buccal; D, distal; I, inferior; L, lingual; M, mesial; O, occlusal. Scale bar is 1 cm. (TIF) [file pone.0067233.s010.tif]

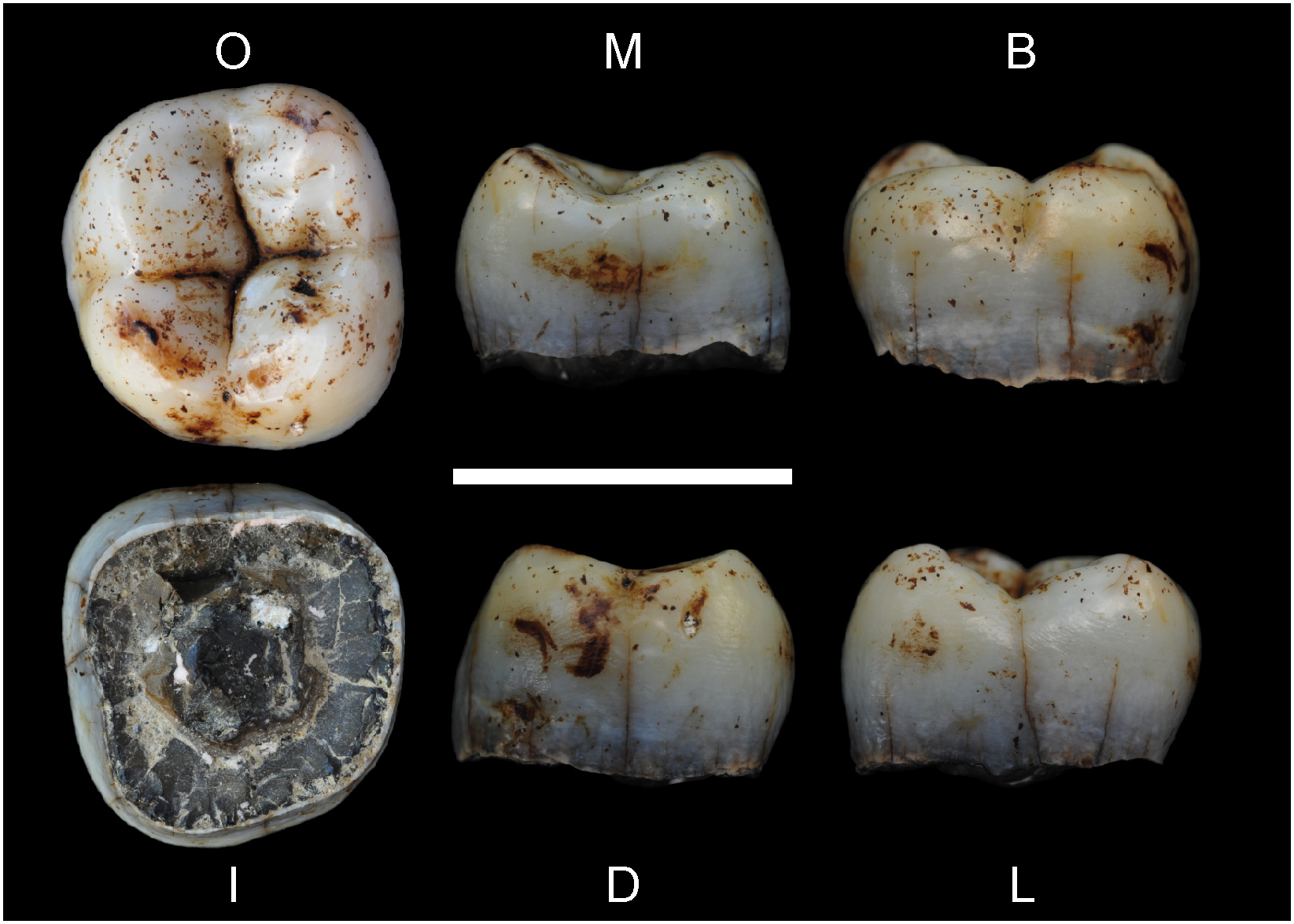

Supplement: Figure S11 — The specimen PCG09_KII_Z:1.37. B, buccal; D, distal; I, inferior; L, lingual; M, mesial; O, occlusal. Scale bar is 1 cm. (TIF) [file pone.0067233.s011.tif]

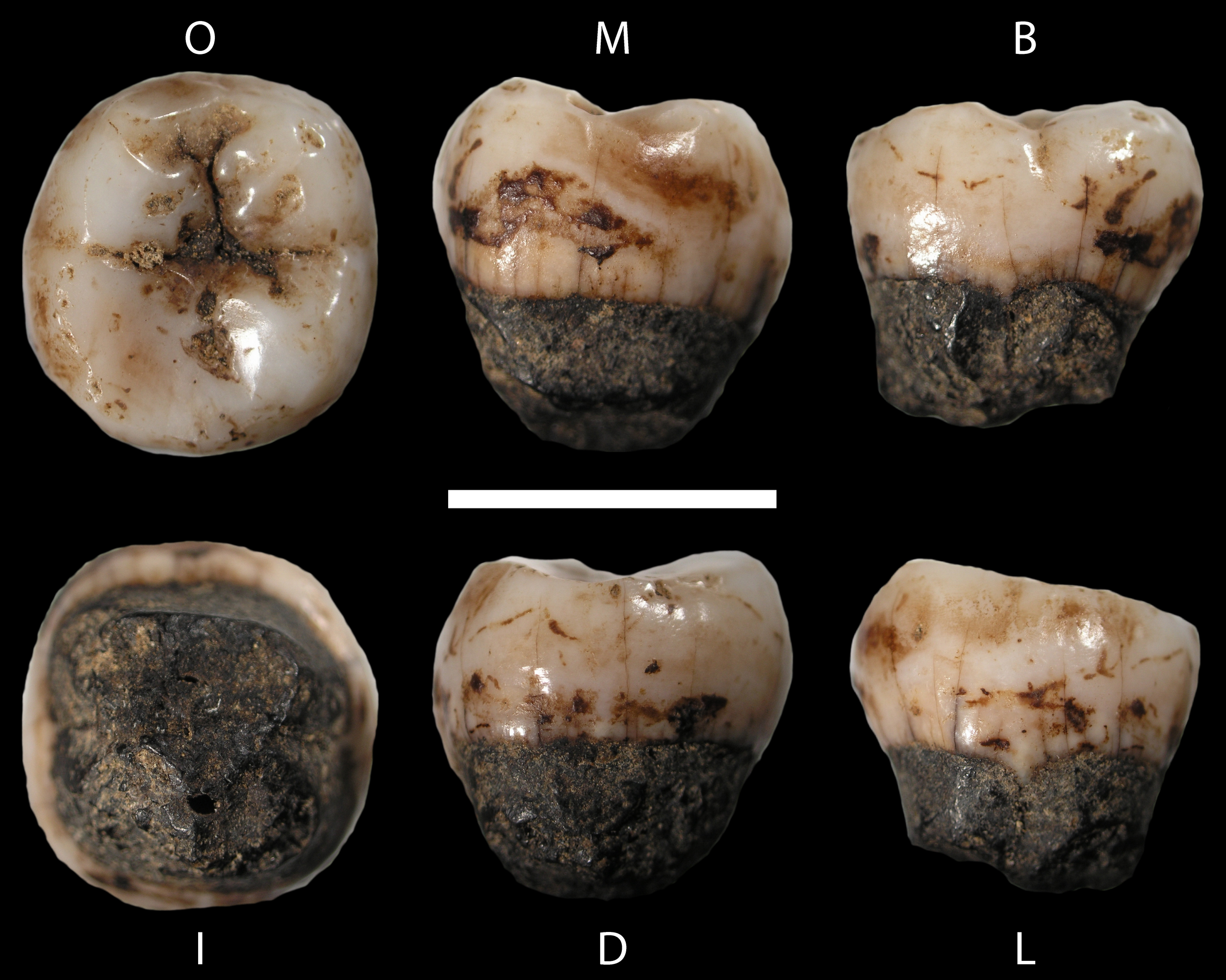

Supplement: Figure S12 — The specimenNG92.3. B, buccal; D, distal; I, inferior; L, lingual; M, mesial; O, occlusal. Scale bar is 1 cm. (TIF) [file pone.0067233.s012.tif]

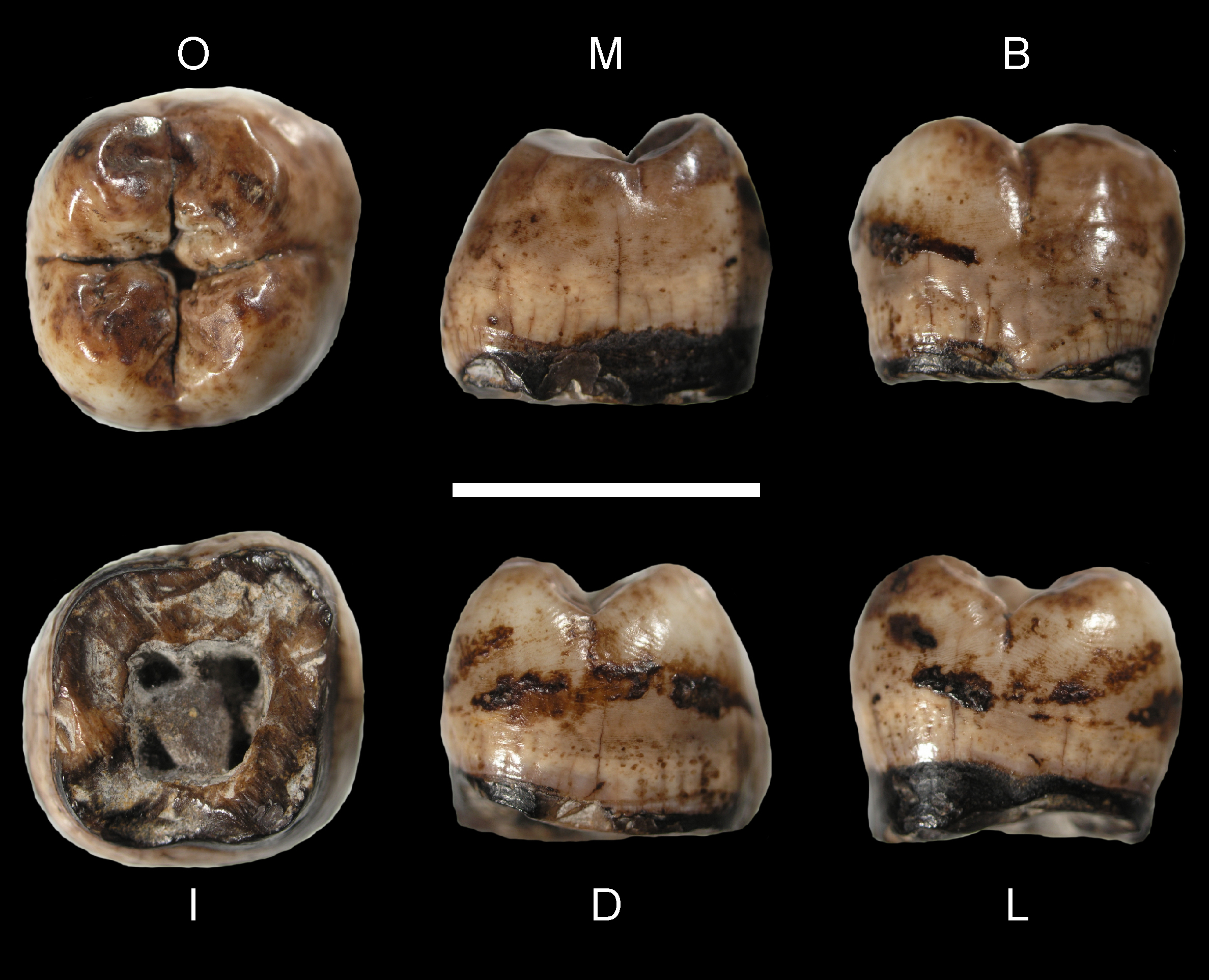

Supplement: Figure S13 — The specimen NG92 D6 ZE 57 s/d 76. B, buccal; D, distal; I, inferior; L, lingual; M, mesial; O, occlusal. Scale bar is 1 cm. (TIF) [file pone.0067233.s013.tif]

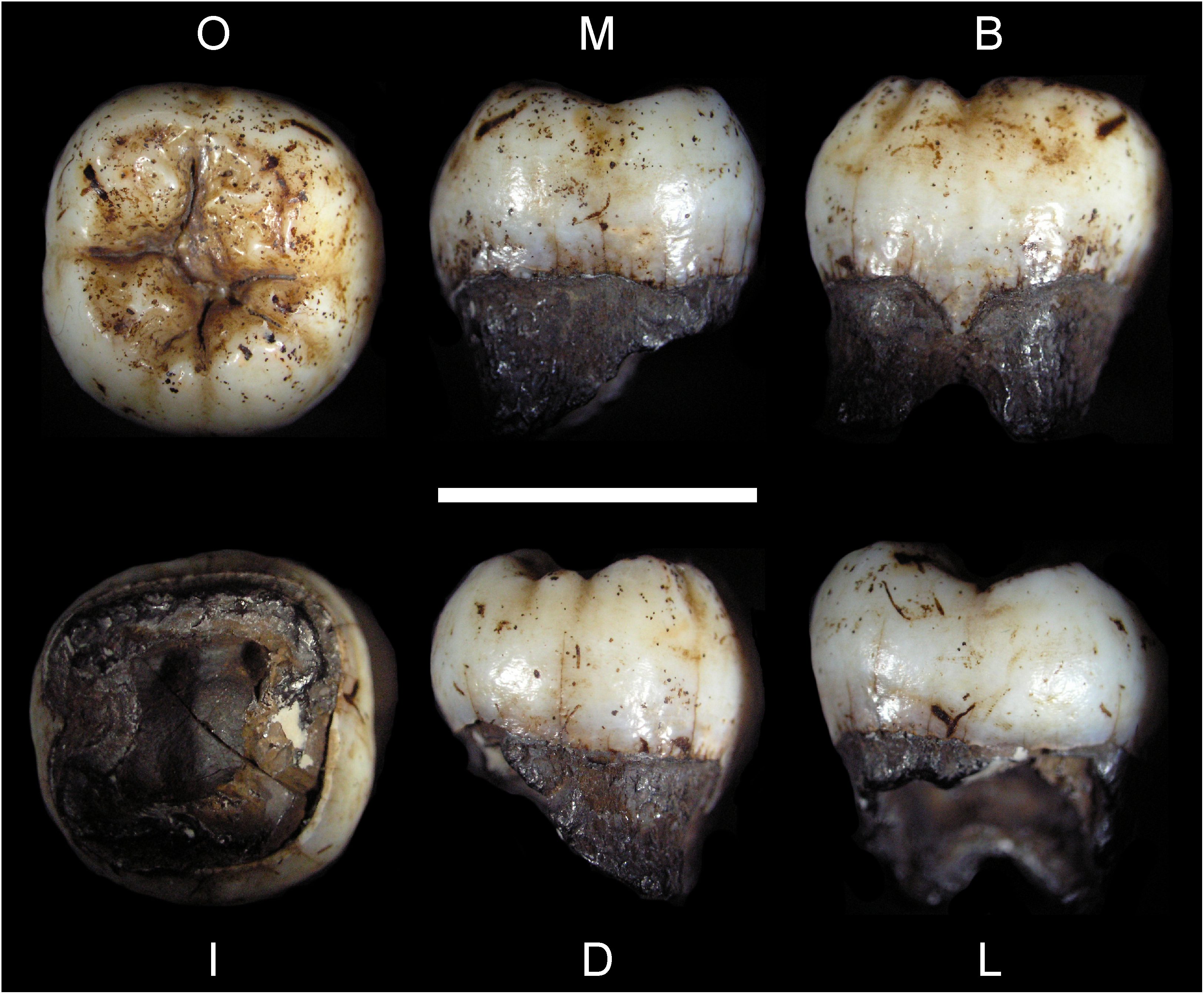

Supplement: Figure S14 — The specimenNG0802.2. B, buccal; D, distal; I, inferior; L, lingual; M, mesial; O, occlusal. Scale bar is 1 cm. (TIF) [file pone.0067233.s014.tif]

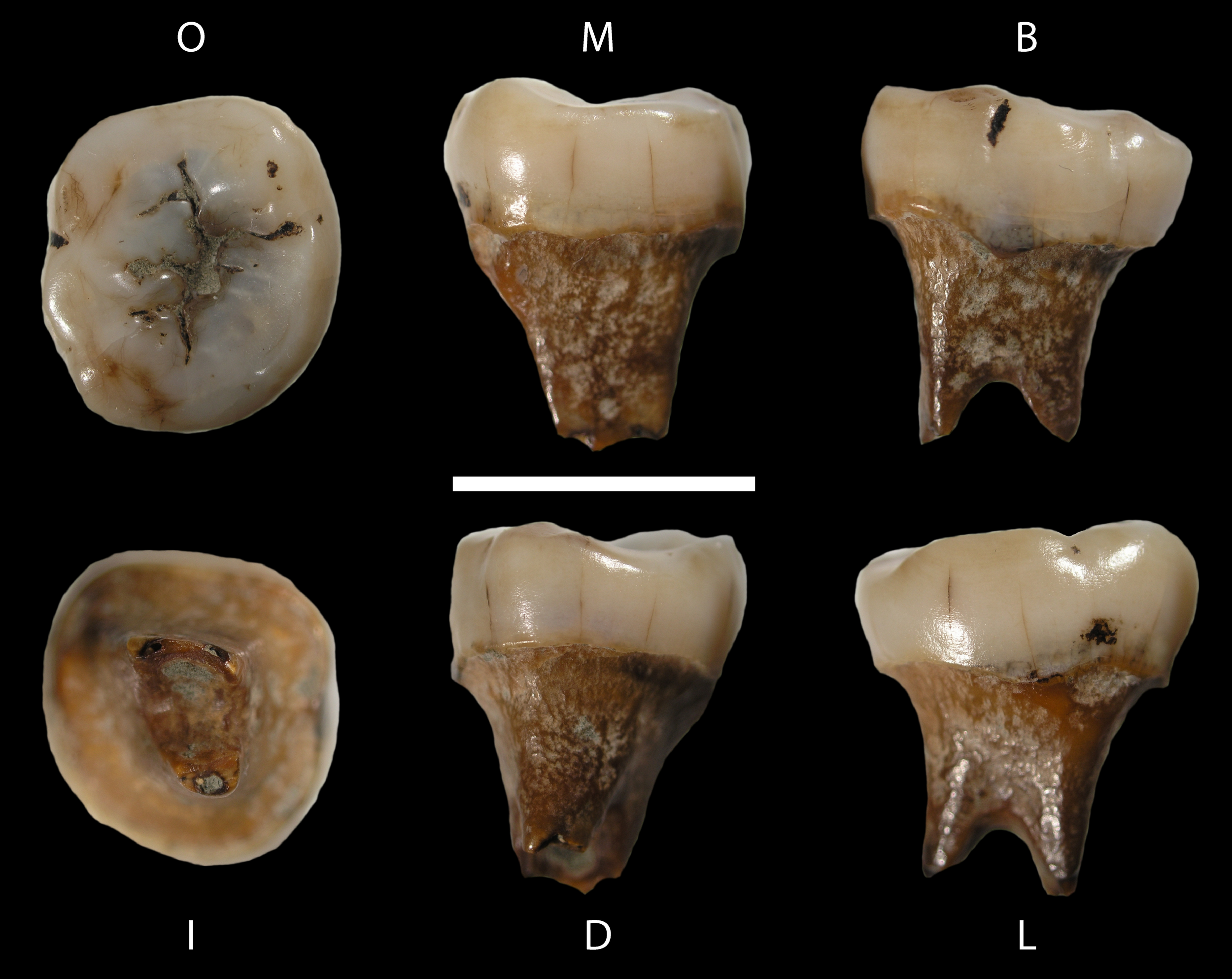

Supplement: Figure S15 — The specimen NG9107.2. B, buccal; D, distal; I, inferior; L, lingual; M, mesial; O, occlusal. Scale bar is 1 cm. (TIF) [file pone.0067233.s015.tif]
