# Supplementary material for: Additional Evidence for Morpho-Dimensional Tooth Crown Variation in a New Indonesian H. erectus Sample from the Sangiran Dome (Central Java)
Source: PLoS One. 2013 Jul 3;8(7):e67233. doi: 10.1371/journal.pone.0067233 (PMC3700995; doi:10.1371/journal.pone.0067233)
Supplement: Table S1 — The comparative dental record used for the assessment of crown size, Hld development, occlusal groove pattern, and cusp proportions. (DOC) [file pone.0067233.s016.doc]

Table S1. The comparative dental record used for the assessment of crown size, Hld development, occlusal groove pattern, and cusp proportions.

|  | **crown diameters** | | | **Hld development and groove pattern** | | **cusp proportions** | |
| --- | --- | --- | --- | --- | --- | --- | --- |
|  | **LM1** | **LM2** | **LM3** | **LM2** | **LM3** | **LM2** | **LM3** |
| ***H. habilis/rudolfensis* (HHR)** | n=12 | n=12 | n=9 | n=4 | n=3 | n=7 | n=6 |
| KNM-ER 1480^a^ |  |  | x |  |  |  | x |
| KNM-ER 1482^b^ | x | x | x |  |  |  |  |
| KNM-ER 1506^a^ | x | x |  |  |  | x |  |
| KNM-ER 1507^a^ | x |  |  |  |  |  |  |
| KNM-ER 1590^a^ |  | x |  |  |  | x |  |
| KNM-ER 1801^a,b^ | x |  | x |  |  |  |  |
| KNM-ER 1802^a,c^ | x | x |  | x | x | x |  |
| KNM-ER 1805^a^ |  | x | x |  |  |  | x |
| KNM-ER 3734^a^ | x | x |  |  |  |  |  |
| KNM-ER 60000^b^ | x | x | x |  |  |  |  |
| OH 4^a^ |  |  | x |  |  |  | x |
| OH 7^a,c^ | x | x |  | x |  | x |  |
| OH 13^a,c^ | x | x | x | x | x | x | x |
| OH 16^a,c^ | x | x | x | x | x | x | x |
| OH 27^a^ |  |  | x |  |  |  | x |
| OH 37^a^ | x | x |  |  |  |  |  |
| UR-501^d,e^ | x | x |  |  |  | x |  |
| ***H. erectus* from East Africa (HEA)** | n=5 | n=5 | n=4 | n=5 | n=1 | n=3 | n=5 |
| KNM-ER 730^a^ |  |  | x |  |  |  |  |
| KNM-ER 806^a,c^ | x | x | x | x |  | x | x |
| KNM-ER 820^a^ | x |  |  |  |  |  |  |
| KNM-ER 992^a,c^ | x | x | x | x |  | x | x |
| KNM-ER 1808^a,c^ |  | x |  | x |  |  | x |
| KNM-ER 1812^a^ |  |  | x |  |  |  | x |
| KNM-WT 8556^c^ |  |  |  |  | x |  |  |
| KNM-WT 15000^a,c^ | x | x |  | x |  |  |  |
| OH 22^a,c^ | x | x |  | x |  | x | x |
| ***H. erectus* from Georgia (HEG)** | n=2 | n=3 | n=2 | n=2 | n=1 |  |  |
| D211^f,c^ | x | x |  | x | x |  |  |
| D2600^f,c^ |  | x | x |  |  |  |  |
| D2735^f^ | x | x | x | x |  |  |  |
| ***H. antecessor* (HA)** | n=2 | n=2 | n=1 | n=2 | n=2 | n=1 |  |
| ATD-6-5^c,e^ | x | x |  | x | x | x |  |
| ATD-6-96^c,e^ | x | x | x | x | x |  |  |
| **Robust hominins from Java (RHJ)** | n=1 | n=4 | n=2 | n=3 | n=1 |  |  |
| Bk 7905^g^ |  | x |  | x |  |  |  |
| Sangiran 5^a^ | x | x |  |  |  |  |  |
| Sangiran 6b^h^ |  |  | x | x |  |  |  |
| Sangiran 9^g^ |  | x | x | x | x |  |  |
| Sangiran 33^i^ |  | x |  |  |  |  |  |
| **Javanese *H. erectus* (HEJ)** | n=11 | n=10 | n=4 | n=7 | n=5 | n=3 | n=1 |
| Arjuna 8^i^ |  | x |  |  |  |  |  |
| Ng 8503^g^ | x | x |  | x |  |  |  |
| Sangiran 1b^a,j,k^ | x | x |  | x | x | x | x |
| Sangiran 7-20^h^ | x |  |  |  |  |  |  |
| Sangiran 7-42^h^ | x |  |  |  |  |  |  |
| Sangiran 7-43^h^ | x |  |  |  |  |  |  |
| Sangiran 7-61^h^ | x |  |  |  |  |  |  |
| Sangiran 7-62^h^ | x |  |  |  |  |  |  |
| Sangiran 7-64^h,k^ |  | x |  | x |  | x |  |
| Sangiran 7-65^h,k^ |  | x |  | x |  | x |  |
| Sangiran 7-76^h^ | x |  |  |  |  |  |  |
| Sangiran 7-78^h^ | x |  |  |  |  |  |  |
| Sangiran 7-84^h^ |  | x |  | x |  |  |  |
| Sangiran 8^g^ |  |  | x |  | x |  |  |
| Sangiran 21^g^ |  |  | x |  | x |  |  |
| Sangiran 22^g^ | x | x | x | x | x |  |  |
| Sangiran 24^i^ |  | x |  |  |  |  |  |
| Sangiran 37^i^ |  | x |  |  |  |  |  |
| Sb 8103^g^ | x | x | x | x | x |  |  |
| **Chinese *H. erectus* (HEC)** | n=12 | n=8 | n=9 | n=5 | n=4 |  |  |
| ZKD A1-1^a^ | x |  |  |  |  |  |  |
| ZKD A2-2^a^ | x | x | x |  | x |  |  |
| ZKD A3-56^a^ | x |  |  |  |  |  |  |
| ZKD AN-518^a^ |  | x |  |  |  |  |  |
| ZKD B1-3^a^ | x |  |  |  |  |  |  |
| ZKD B1-63^a^ |  | x |  | x |  |  |  |
| ZKD B2-64^a^ |  |  |  |  | x |  |  |
| ZKD B3-9^a^ | x |  |  |  |  |  |  |
| ZKD B4-75^a^ |  | x |  | x |  |  |  |
| ZKD C1-4^a^ |  |  |  | x |  |  |  |
| ZKD C3-45^a^ | x |  |  |  |  |  |  |
| ZKD D1-43^a^ |  | x |  | x |  |  |  |
| ZKD D1-61^a^ |  |  | x |  |  |  |  |
| ZKD F1-5^a^ | x |  |  | x |  |  |  |
| ZKD F1-25^a^ |  |  | x |  | x |  |  |
| ZKD G1-6^a^ | x | x | x |  | x |  |  |
| ZKD G1-7^a^ |  |  | x |  |  |  |  |
| ZKD H1-12^a^ |  |  | x |  |  |  |  |
| ZKD I1-PA87^a^ | x |  |  |  |  |  |  |
| ZKD K1-96^a^ | x | x |  |  |  |  |  |
| ZKD L4-302^a^ |  |  | x |  |  |  |  |
| ZKD L4-309^a^ | x |  |  |  |  |  |  |
| ZKD M1-308^a^ |  |  | x |  |  |  |  |
| ZKD M3-305^a^ |  |  | x |  |  |  |  |
| ZKD M3-310^a^ |  | x |  |  |  |  |  |
| ZKD O2-314^a^ | x |  |  |  |  |  |  |
| **North African *H. heidelbergensis* (HHNA)** | n=3 | n=3 | n=3 | n=3 | n=4 | n=3 | n=3 |
| Rabat^c^ |  |  |  |  | x |  |  |
| Tighenif 1^a,c,k^ | x | x | x | x | x | x | x |
| Tighenif 2^a,c,k^ | x | x | x | x | x | x | x |
| Tighenif 3^a,c,k^ | x | x | x | x | x | x | x |
| **European *H. heidelbergensis* (HHE)** | n=8 | n=9 | n=6 | n=28 | n=26 |  |  |
| Arago 13^c^ |  |  |  | x | x |  |  |
| Arago 68^c^ |  |  |  | x |  |  |  |
| Arago 69^c^ |  |  |  | x |  |  |  |
| AT-169^l^ |  |  | x | n=21 | n=22 |  |  |
| AT-222^l^ |  |  | x |  |  |
| AT-250^l^ |  | x |  |  |  |
| AT-271^l^ |  | x |  |  |  |
| AT-272^l^ | x |  |  |  |  |
| AT-273^l^ |  | x |  |  |  |
| AT-284^l^ |  | x |  |  |  |
| AT-285^l^ | x |  |  |  |  |
| AT-286^l^ | x |  |  |  |  |
| AT-300^l^ | x | x | x |  |  |
| AT-505^l^ |  | x | x |  |  |
| AT-556^l^ | x |  |  |  |  |
| AT-557^l^ |  | x |  |  |  |
| AT-561^l^ | x |  |  |  |  |
| Mauer^m,c^ | x | x | x | x | x |  |  |
| Montmaurin^n,c^ | x | x | x | x | x |  |  |
| Pontneywydd^c^ |  |  |  | x | x |  |  |
| **Neanderthals (NEA)** | n=5 | n=11 | n=7 | n=29 | n=23 |  |  |
| Fondo Cattie^c^ |  |  |  |  | x |  |  |
| Fosselone^n^ | x |  |  |  |  |  |  |
| Gibraltar 2^c^ |  |  |  | x |  |  |  |
| Grotte Boccard^n^ |  | x |  |  |  |  |  |
| Hortus^c^ |  |  |  | x | x |  |  |
| Külna 1^c^ |  |  |  | x | x |  |  |
| Kebara^n,c^ |  | x | x | x |  |  |  |
| Krapina^c^ |  |  |  | x | x |  |  |
| Lakonis I^n^ |  |  | x |  |  |  |  |
| La Quina^n^ | x | x | x |  |  |  |  |
| Le Moustier^c^ |  |  |  | x | x |  |  |
| Montgaudier^n^ | x |  |  |  |  |  |  |
| Petit-Puymoyen^c^ |  |  |  | x |  |  |  |
| Regourdou 1^o^ | x | x | x |  |  |  |  |
| Saint Césaire^c^ |  |  |  | x | x |  |  |
| Shanidar 1^p^ |  | x |  |  |  |  |  |
| Shanidar 2^p^ |  | x |  |  |  |  |  |
| Shanidar 6^p^ |  | x |  |  |  |  |  |
| Shovakh 1^n^ |  |  | x |  |  |  |  |
| Sidrón^c^ |  |  |  | x | x |  |  |
| Tabun^c^ |  |  |  | x | x |  |  |
| Vindija^c^ |  |  |  | x | x |  |  |
| ***H. floresiensis* (HF)** | n=2 | n=2 | n=2 |  |  |  |  |
| LB1^q,r^ | x | x | x |  |  |  |  |
| LB6^q,r^ | x | x | x |  |  |  |  |
| **Fossil modern humans (FMH)** | n=79 | n=80 | n=54 |  |  |  |  |
| Abri Blanchard^n^ |  |  | x |  |  |  |  |
| Abri Pataud^n^ |  | x | x |  |  |  |  |
| Arene Candide^n^ |  | x | x |  |  |  |  |
| Barma Grande^n^ |  | x | x |  |  |  |  |
| Cap Blanc^n^ |  | x |  |  |  |  |  |
| Grotte de la Balauzière^n^ | x |  | x |  |  |  |  |
| Grotte des Enfants^n^ | x | x | x |  |  |  |  |
| La Madeleine^n^ | x | x |  |  |  |  |  |
| Les Rois^n^ | x | x | x |  |  |  |  |
| Les Vachons^n^ | x | x |  |  |  |  |  |
| Mas d'Azil^n^ | x | x |  |  |  |  |  |
| Mladeč^n^ | x | x | x |  |  |  |  |
| Qafzeh^n^ | x | x | x |  |  |  |  |
| Paglicci^n^ | x | x |  |  |  |  |  |
| Pavlov^n^ | x | x | x |  |  |  |  |
| Předmost^n^ | x | x | x |  |  |  |  |
| Romanelli^n^ | x | x | x |  |  |  |  |
| Romito^n^ | x | x | x |  |  |  |  |
| Saint Germain La Rivière^n^ | x | x |  |  |  |  |  |
| **Extant humans (EH)** | n=174 | n=204 | n=185 | n=136 | n=81 | n=71 | n=5 |
| Specimens of European origin^e,k,n^ | x | x | x | x | x | x | x |

^a^Wood, 1991;^b^Leakey et al., 2012; ^c^Martinón-Torres, 2006; ^d^Bromage et al., 1995; ^e^Bermúdez de Castro et al., 1999; ^f^Martinón-Torres et al., 2008; ^g^Kaifu et al., 2005a; ^h^Grine and Franzen, 1994; ^i^Widianto, 1993; ^j^von Koenigswald, 1940: ^k^original data; ^l^Bermúdez de Castro, 1993; ^m^Howell, 1960; ^n^anthropologicaldata.free.fr (cf. Voisin et al., 2011); ^o^Maureille et al., 2001; ^p^Alt et al., 2006; ^q^Morwood et al., 2005; ^r^Brown and Maeda, 2009.

**References**

Alt KW, Kaulich B, Reisch L, Vogel H, Rosendahl W. (2006) The Neanderthalian molar from Hunas, Germany. HOMO 57: 187-200.

Bermúdez de Castro JM. (1993) The Atapuerca dental remains. New evidence (1987–1991 excavations) and interpretations. J Hum Evol 24: 339-371.

Bermúdez de Castro JM, Rosas A, Nicolás ME. (1999) Dental remains from Atapuerca-TD6 (Gran Dolina site, Burgos, Spain). J Hum Evol 37: 523-566.

Bromage TG, Schrenk F, Zonneveld FW. (1995) Paleoanthropology of the Malawi Rift: an early hominid mandible from the Chiwondo Beds, Northern Malawi. J Hum Evol 28: 71-108.

Brown P, Maeda T. (2009) Liang Bua *Homo floresiensis* mandibles and mandibular teeth: a contribution to the comparative morphology of a new hominin species. J Hum Evol 57: 571-596.

Grine FE, Franzen JL. (1994) Fossil hominid teeth from the Sangiran dome (Java, Indonesia). Courier Forsch Senckenberg 171: 75-103.

Howell FC. (1960) European and Northwest African Middle Pleistocene hominids. Curr Anthropol 1: 195–232.

Kaifu Y, Aziz F, Baba H. (2005a) Hominid mandibular remains from Sangiran: 1952-1986 collection. Am J Phys Anthropol 128: 497-519.

Leakey MG, Spoor F, Dean MC, Feibel CS, Antón SC, Kiarie C, Leakey LN. (2012) New fossils from Koobi Fora in northern Kenya confirm taxonomic diversity in early *Homo*. Nature 488: 201-204.

Martinón-Torres M. (2006) Evolución del aparato dental en homínidos: estudio de los dientes humanos del Pleistoceno de Sierra de Atapuerca (Burgos). PhD dissertation. University of Santiago de Compostella: Santiago de Compostella.

Martinón-Torres M, Bermúdez de Castro JM, Gómez-Robles A, Margvelshvill A, Prado L, et al. (2008) Dental remains from Dmanisi (Republic of Georgia): morphological analysis and comparative study. J Hum Evol 55: 249-273.

Maureille B, Rougier H, Houet F, Vandermeersch B. (2001) Les dents inférieures du Néandertalien Regourdou 1 (site de Regourdou, commune de Montignac, Dordogne): analyses métriques et comparatives. Paleo 13: 183-200.

Morwood MJ, Brown P, Jatmiko, Sutikna T, Saptomo EW, Westaway KE, Due RA, Roberts RG, Maeda T, Wasisto S, Djubiantono T. (2005) Further evidence for small-bodied hominins from the Late Pleistocene of Flores, Indonesia. Nature 437: 1012-1017.

Voisin JL, Condemi S, Frayer D. (2011) Teeth, internet and a free, new database. Bull Mém Soc Anthropol Paris 23: S39 (abstract).

von Koenigswald GHR. (1940) Neue *Pithecanthropus*-Funde 1936-1938. Wet Meded Dienst Mijnb Ned Oost-Indië 28: 1-223.

Widianto H. (1993) Unité et diversité des hominidés fossiles de Java : présentation de restes humains inédits. PhD dissertation. Paris: MNHN.

Wood BA. (1991) Koobi Fora research project. Vol. 4. Hominid cranial remains from Koobi Fora. Oxford: Clarendon Press.
