# Supplementary material for: Additional Evidence for Morpho-Dimensional Tooth Crown Variation in a New Indonesian H. erectus Sample from the Sangiran Dome (Central Java)
Source: PLoS One. 2013 Jul 3;8(7):e67233. doi: 10.1371/journal.pone.0067233 (PMC3700995; doi:10.1371/journal.pone.0067233)
Supplement: Table S2 — The comparative dental record used for the assessment of relative cusp areas (in %). (DOCX) [file pone.0067233.s017.docx]

Table S2. The comparative dental record used for the assessment of relative cusp areas (in %).

|  |  | **Tooth** | **Prd** | **Med** | **Hyd** | **End** | **Hld** |
| --- | --- | --- | --- | --- | --- | --- | --- |
| ***H. habilis/rudolfensis* (HHR)** | |  |  |  |  |  |  |
|  | OH 7 ^b^ | M_2_ | 25.9 | 22.6 | 19.7 | 15.3 | 16.5 |
|  | OH 13 ^b^ | M_2_ | 24.7 | 21.5 | 19.4 | 19.0 | 15.4 |
|  | OH 16 ^b^ | M_2_ | 26.9 | 21.0 | 21.0 | 13.5 | 17.6 |
|  | KNM-ER 1506 ^b^ | M_2_ | 23.6 | 21.3 | 22.3 | 16.7 | 16.1 |
|  | KNM-ER 1590 ^b^ | M_2_ | 23.5 | 21.4 | 24.0 | 14.8 | 16.3 |
|  | KNM-ER 1802 ^b^ | M_2_ | 24.2 | 22.9 | 22.9 | 15.1 | 15.1 |
|  | KNM-ER 1805 ^b^ | M_3_ | 24.6 | 24.9 | 19.1 | 16.2 | 15.2 |
|  | UR-501 ^b^ | M_2_ | 21.4 | 21.5 | 19.9 | 15.2 | 22.0 |
|  | OH 4 ^b^ | M_3_ | 24.3 | 24.6 | 17.4 | 16.8 | 16.8 |
|  | OH 13 ^b^ | M_3_ | 23.2 | 21.8 | 17.3 | 19.7 | 18.0 |
|  | OH 13 ^b^ | M_3_ | 21.3 | 24.4 | 16.0 | 20.9 | 17.4 |
|  | OH 16 ^b^ | M_3_ | 28.1 | 26.2 | 16.4 | 14.2 | 15.0 |
|  | OH 16 ^b^ | M_3_ | 27.6 | 23.8 | 17.9 | 13.8 | 16.8 |
|  | OH 27 ^b^ | M_3_ | 25.3 | 25.6 | 15.6 | 14.7 | 18.8 |
| ***H. erectus* from East Africa (HEA)** | |  |  |  |  |  |  |
|  | OH 22^b^ | M_2_ | 31.8 | 24.7 | 21.2 | 9.0 | 13.3 |
|  | KNM-ER 806 ^b^ | M_2_ | 23.4 | 20.8 | 21.5 | 17.3 | 17.0 |
|  | KNM-ER 806 ^b^ | M_3_ | 23.8 | 19.9 | 15.9 | 17.9 | 22.5 |
|  | KNM-ER 806 ^b^ | M_3_ | 22.7 | 23.8 | 18.6 | 16.0 | 19.0 |
|  | KNM-ER 992 ^b^ | M_2_ | 27.0 | 24.3 | 20.5 | 15.6 | 12.5 |
|  | KNM-ER 992 ^b^ | M_2_ | 26.6 | 25.9 | 19.8 | 15.1 | 12.6 |
|  | KNM-ER 992 ^b^ | M_3_ | 30.6 | 23.4 | 16.1 | 12.1 | 17.7 |
|  | KNM-ER 992 ^b^ | M_3_ | 27.6 | 25.7 | 18.3 | 10.1 | 18.3 |
|  | KNM-ER 1480 ^b^ | M_3_ | 21.4 | 27.3 | 17.2 | 18.8 | 15.3 |
|  | KNM-ER 1808 ^b^ | M_2_ | 24.2 | 22.7 | 20.0 | 16.5 | 16.5 |
|  | KNM-ER 1812 ^b^ | M_3_ | 22.8 | 19.2 | 21.0 | 19.2 | 17.8 |
| ***H. antecessor* (HA)** | |  |  |  |  |  |  |
|  | TD6 H1 | M_2_ | 25.5 | 15.0 | 25.4 | 20.5 | 13.5 |
| **Javanese *H. erectus* (HEJ)** | |  |  |  |  |  |  |
|  | Sangiran 1b | M_2_ | 24.3 | 26.9 | 21.9 | 14.9 | 12.1 |
|  | Sangiran 1b | M_3_ | 25.1 | 22.6 | 19.4 | 17.1 | 15.8 |
|  | Sangiran 7-64 | M_2_ | 27.1 | 24.9 | 17.1 | 16.6 | 14.4 |
|  | Sangiran 7-65 | M_2_ | 32.0 | 26.7 | 17.0 | 16.1 | 8.2 |
| **North African *H. heidelbergensis* (HHNA)** | |  |  |  |  |  |  |
|  | Tighenif 1 | M_2_ | 30.0 | 24.8 | 26.3 | 12.7 | 6.3 |
|  | Tighenif 1 | M_3_ | 37.4 | 27.5 | 14.4 | 10.2 | 10.5 |
|  | Tighenif 2 | M_2_ | 26.6 | 23.9 | 19.6 | 20.1 | 9.8 |
|  | Tighenif 2 | M_3_ | 27.7 | 30.1 | 13.6 | 17.2 | 11.4 |
|  | Tighenif 3 | M_2_ | 27.5 | 24.7 | 21.2 | 15.9 | 10.6 |
|  | Tighenif 3 | M_3_ | 26.3 | 24.8 | 19.5 | 19.2 | 10.2 |
| **Extant humans (EH)** | |  |  |  |  |  |  |
|  | Extant humans mean (N=71)^a^ | M_2_ | 27.4 | 20.4 | 21.0 | 19.9 | 11.2 |
|  | EH1 | M_3_ | 24.8 | 22.1 | 13.0 | 22.3 | 17.8 |
|  | EH2 | M_3_ | 27.4 | 23.8 | 23.0 | 18.5 | 7.3 |
|  | EH3 | M_3_ | 25.4 | 22.3 | 11.0 | 25.5 | 15.7 |
|  | EH4 | M_3_ | 27.1 | 22.9 | 18.8 | 23.4 | 7.8 |
|  | EH5 | M_3_ | 20.9 | 23.2 | 20.8 | 21.2 | 13.9 |

^a^Bermúdez de Castro et al., 1999; ^b^Wood, 1991

**References**

Bermúdez de Castro JM, Rosas A, Nicolás ME. (1999) Dental remains from Atapuerca-TD6 (Gran Dolina site, Burgos, Spain). J Hum Evol 37: 523-566.

Wood BA. (1991) Koobi Fora research project. Vol. 4. Hominid cranial remains from Koobi Fora. Oxford: Clarendon Press.
